# Cryo-EM structure of the *Shigella* type III needle complex

**DOI:** 10.1371/journal.ppat.1008263

**Published:** 2020-02-24

**Authors:** Michele Lunelli, Antje Kamprad, Jörg Bürger, Thorsten Mielke, Christian M. T. Spahn, Michael Kolbe

**Affiliations:** 1 Department of Structural Infection Biology, Centre for Structural Systems Biology (CSSB), Helmholtz-Centre for Infection Research (HZI), Hamburg, Germany; 2 Structural Systems Biology Group, Max Planck Institute for Infection Biology, Berlin, Germany; 3 Institute for Medical Physics and Biophysics, Charité Universitätsmedizin Berlin, Berlin, Germany; 4 Max Planck Institute for Molecular Genetics, Berlin, Germany; 5 Faculty of Mathematics, Informatics and Natural Sciences, University of Hamburg, Hamburg, Germany; Gifu University, JAPAN

## Abstract

The Type III Secretion Systems (T3SS) needle complex is a conserved syringe-shaped protein translocation nanomachine with a mass of about 3.5 MDa essential for the survival and virulence of many Gram-negative bacterial pathogens. This system is composed of a membrane-embedded basal body and an extracellular needle that deliver effector proteins into host cells. High-resolution structures of the T3SS from different organisms and infection stages are needed to understand the underlying molecular mechanisms of effector translocation. Here, we present the cryo-electron microscopy structure of the isolated *Shigella* T3SS needle complex. The inner membrane (IM) region of the basal body adopts 24-fold rotational symmetry and forms a channel system that connects the bacterial periplasm with the export apparatus cage. The secretin oligomer adopts a heterogeneous architecture with 16- and 15-fold cyclic symmetry in the periplasmic N-terminal connector and C-terminal outer membrane ring, respectively. Two out of three IM subunits bind the secretin connector via a β-sheet augmentation. The cryo-EM map also reveals the helical architecture of the export apparatus core, the inner rod, the needle and their intervening interfaces.

## Author summary

Diarrheal diseases evoke about 2.2. million dead people annually and are the second leading cause of postneonatal child mortality worldwide. *Shigella* causing dysentery utilizes the type 3-secretion system (T3SS) to inject virulence factors into the gut cells. The T3SS needle complex is a syringe-shaped nanomachine consisting of two membrane-embedded ring systems that sheath a central export apparatus and a hollow needle-like structure through which the virulence factors are transported. We present here the structure of the *Shigella* T3SS needle complex obtained by high-end electron microscopy. The outer membrane (OM) ring system adopts a mixed 15- and 16-fold cyclic symmetry and the near-atomic structure shows the connection of the inner membrane (IM) and OM rings. Conserved channels in the IM ring connect the bacterial periplasm with the central export apparatus. Similar to the *Salmonella* flagellar system, the export apparatus and its connected needle-like structure assemble in a helical manner. This study advances our understanding of the role of essential structural elements in the T3SS assembly and function.

## Introduction

Bacterial diarrheal diseases cause million deaths in children under age five worldwide [1]. *Shigella* causes dysentery, a disease that accounts for approximately half a million infant deaths annually [2]. Common to many bacterial enteropathogens, *Shigella* colonize the gastrointestinal tract by delivering virulence factors called effectors in a time-dependent fashion to the target cell [3]. Together with *Salmonella* spp and *Yersinia* spp, *Shigella* strains belong to the phylogenetic family of *Salmonella* pathogenic island– 1 (SPI-1) bacteria [4], which employ a Type III Secretion System (T3SS), also named injectisome, to trigger the early phases of cell invasion.

The T3SS structural core is a membrane-embedded transporter called needle complex, which is an evolutionary conserved syringe-like multiprotein assembly with a molecular weight of about 3.5 MDa. This complex is made of a basal body and a hollow needle that extends from the bacterial surface. The needle is a homo-oligomeric structure of different length depending on the species, formed by a helical arrangement of a single small protein of 9 kDa, MxiH in *Shigella* [5,6]. The basal body is assembled by the stepwise incorporation of multiple copies of inner- (MxiG and MxiJ in *Shigella*) and outer- (MxiD in *Shigella*, which belongs to the family of the secretins) membrane proteins to form their respective rings in the corresponding membranes and periplasm [5,7]. These rings embrace the so-called inner rod and export apparatus core. The inner membrane (IM) ring consists of a periplasmic, a transmembrane and a cytoplasmic region. The secretin MxiD forms two rings, namely the periplasmic connector and the outer membrane (OM) pore. The inner rod, which is embedded in the secretin ring system and is formed by MxiI in *Shigella* [8], functions as a conduit that connects the export apparatus and the needle filament. Its exact position in the *Shigella* needle complex and dimension, however, is still unclear. The export apparatus is thought to serve as entry gate for unfolded substrates in the cytoplasm and an anchor for the inner rod in the periplasm [9,10]. The structurally best characterized export apparatus from *Salmonella* consists of five membrane associated proteins [10,11]. Its *Shigella* needle complex orthologues are SpaPQRS (also called Spa24, Spa9, Spa29 and Spa40, respectively) and MxiA. On the cytoplasmic side of the needle complex, a set of different proteins assemble together to form the sorting platform, a complex that recruits, unfolds and delivers T3SS substrates to the cytoplasmic components of the export apparatus [12,13]. Effector proteins targeted to the needle complex are then translocated across the IM through the export apparatus, gliding through the inner rod and the needle before they reach the extracellular space or the host cell [14]. The sorting platform ATPase seems to be important for secretion of T3SS substrates [15]. Several studies suggest that efficient protein translocation requires ATP hydrolysis and a proton motive force [16–19], but the underlying molecular mechanisms are yet to be discovered.

Here, we report the near-atomic structure of the *Shigella* T3SS IM periplasmic ring and of the N-terminal domains of the secretin, revealing their interface and a network of channels in the IM ring, based on high-resolution cryo-EM symmetrized maps obtained by single-particle analysis (3.6 Å and 3.9 Å, respectively). Further, the unsymmetrized map at 5.1 Å resolution of the basal body reveals a mixed C15/C16 symmetry of the secretin oligomer and provides a model for the architecture of the export apparatus core, the inner rod and the start of the needle filament. Altogether, our obtained maps and molecular models provide new insights into the structure and assembly of the *Shigella* needle complex and allow a comparative analysis with the well-studied orthologous complex from *Salmonella*.

## Results

### Reconstruction of the needle complex

**Fig 1:**
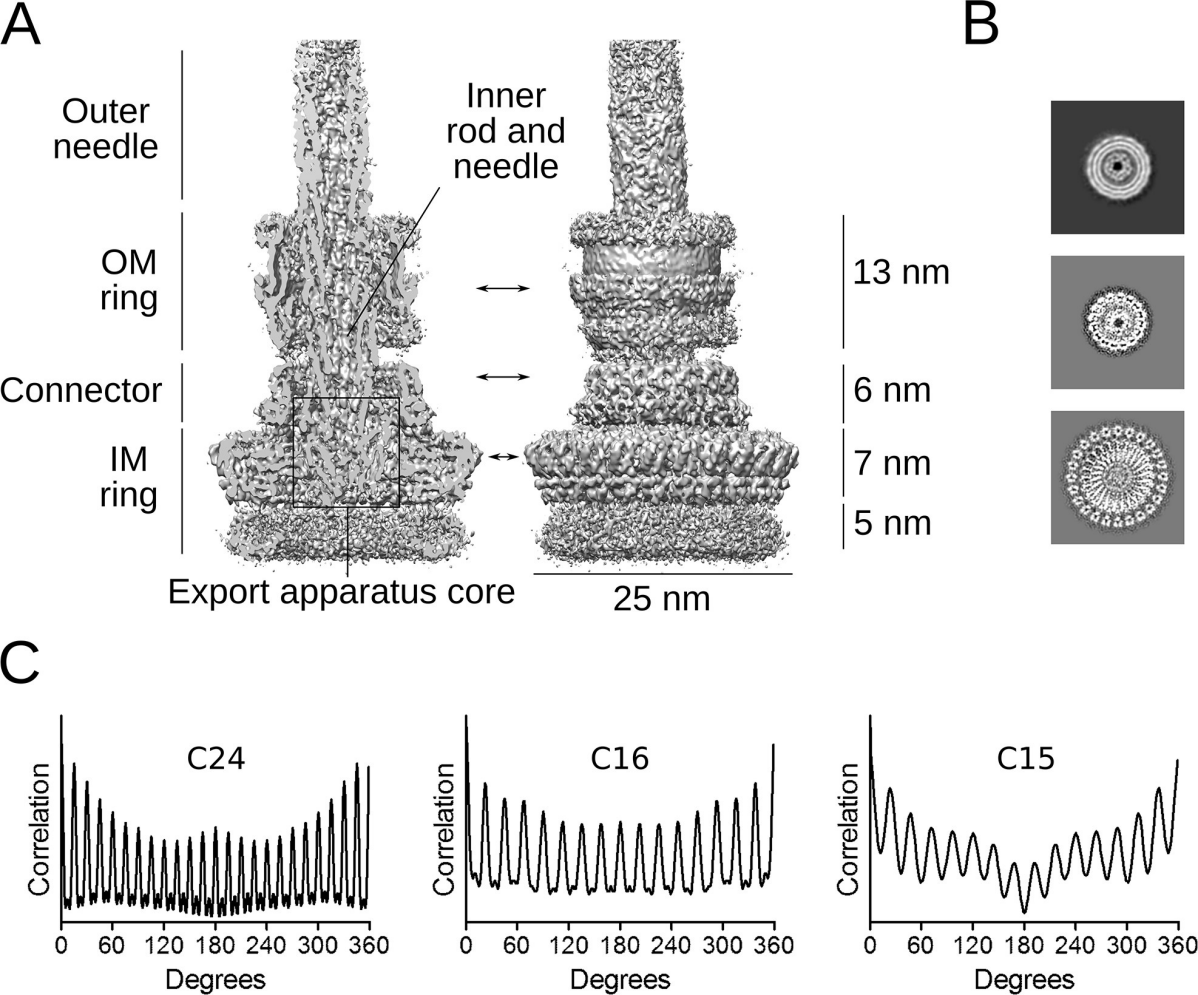
Needle complex reconstruction and symmetry analysis. (**A**) Cross-section and side view of the symmetry-free (C1) reconstruction of the *Shigella* needle complex. The structural features and their dimensions are indicated. Arrows show the position of the slices shown in panel B. (**B**) Horizontal slices of the periplasmic IM ring (bottom panel),the connector (middle panel) and the OM ring (upper panel, from non-sharpened map). (**C**) Rotational autocorrelation analysis of the three slices shown in panel B indicating C24 symmetry for the IM ring (24 peaks spaced by 15 degrees), C16 symmetry for the connector (16 peaks spaced by 22.5 degrees) and C15 symmetry for the OM ring (15 peaks spaced by 24 degrees). A circular mask with inner radius 55 Å and outer radius 76 Å was applied to the OM ring slice to highlight the 15-fold periodicity.

We aimed at obtaining high-resolution structural data of the *Shigella* T3SS needle complex by single particle cryo-EM analysis. For this, we imaged vitrified needle complexes with a 300 kV FEI Titan Krios microscope in combination with a Falcon II direct electron detector. 104,272 particle images obtained after 2D classification were subjected to 3D classification in three classes. The main class included 69% of the particles, which we used for high-resolution refinement (Fig 1 and S1 Fig).

The 3D reconstruction of the intact *Shigella* needle complex without imposing symmetry (C1) yielded a map with an overall resolution of 5.1 Å (Table 1), as determined by the gold-standard Fourier shell correlation (FSC) value of 0.143 (S2A Fig) [20]. The basal body of the *Shigella* needle complex measures approximately 31 nm in length and is tapering from 25 nm diameter in the IM ring down to 15 nm in the outer membrane (OM) ring (Fig 1A). In our map, IM and OM rings, connector, inner rod, needle and export apparatus core are distinguishable. The OM ring and the needle appear slightly tilted with respect to the vertical axis of the rest of the basal body. The periplasmic IM ring and the connector regions show the highest local resolution in the range of 3.5 to 5 Å (S3A Fig). The map reveals their rotational symmetry by slicing through the vertical axis (Fig 1B and 1C). The IM ring, the connector and the OM ring show 24, 16 and 15 modulations, respectively, indicating that these regions have corresponding cyclic symmetries C24, C16 and C15. Our observations are consistent with previous studies showing heterogeneous symmetries in the T3SS needle complex [6,21,22]. The symmetries of the basal body ring systems are conserved with the *Salmonella* T3SS; in particular the OM ring adopts a different cyclic symmetry than the connector as reported in a recent paper [23].

**Table 1:** Cryo-EM data collection, refinement and validation statistics.

**Map**	**IM ring**	**Connector & IM ring**	**Full NC**
EMDB ID	EMD-10045	EMD-10040	EMD-10046
Magnification	100,000	100,000	100,000
Voltage (kV)	300	300	300
Electron exposure (e^−^ Å^−2^)	25	25	25
Defocus range (μm)	1.5–4	1.5–4	1.5–4
Pixel size (Å)	1.38	1.38	1.38
Symmetry imposed	C24	C8	C1
Initial particle images (no.)	171,833	171,833	171,833
Final particle images (no.)	72,298	72,298	72,298
Map resolution (Å)	3.6	3.9	5.1
FSC threshold	0.143	0.143	0.143
Map sharpening *B* factor	-128	-120	-162
**Model**	**IM periplasmic ring** *	Connector **	Export Apparatus and inner rod ***
PDB ID	6RWX	6RWK	6RWY
Model resolution (Å)	3.5	3.7	8.0
FSC threshold	0.5	0.5	0.5
Model composition			
Non-hydrogen atoms	70,968	21,760	22,677
Protein residues	8,784	2,688	3,160
R.m.s. deviations			
Bond lengths (Å)	0.009	0.007	0.004
Bond angles (°)	1.17	0.75	0.83
MolProbity score	1.62	2.84	2.78
Clashscore	3.35	14.06	19.58
Poor rotamers (%)	0.00	5.83	7.11
Ramachandran plot			
Favored (%)	91.71	87.80	94.98
Allowed (%)	8.29	12.20	4.76
Disallowed (%)	0.00	0.00	0.26

*) MxiG_152-340_ and MxiJ_21-197_

**) MxiD_34-171_ & MxiG_338-367_

***) SpaP_6-213_, SpaQ_1-86_, SpaR_17-256_, inner rod subunits and MxiH_11-83_

**Fig 2:**
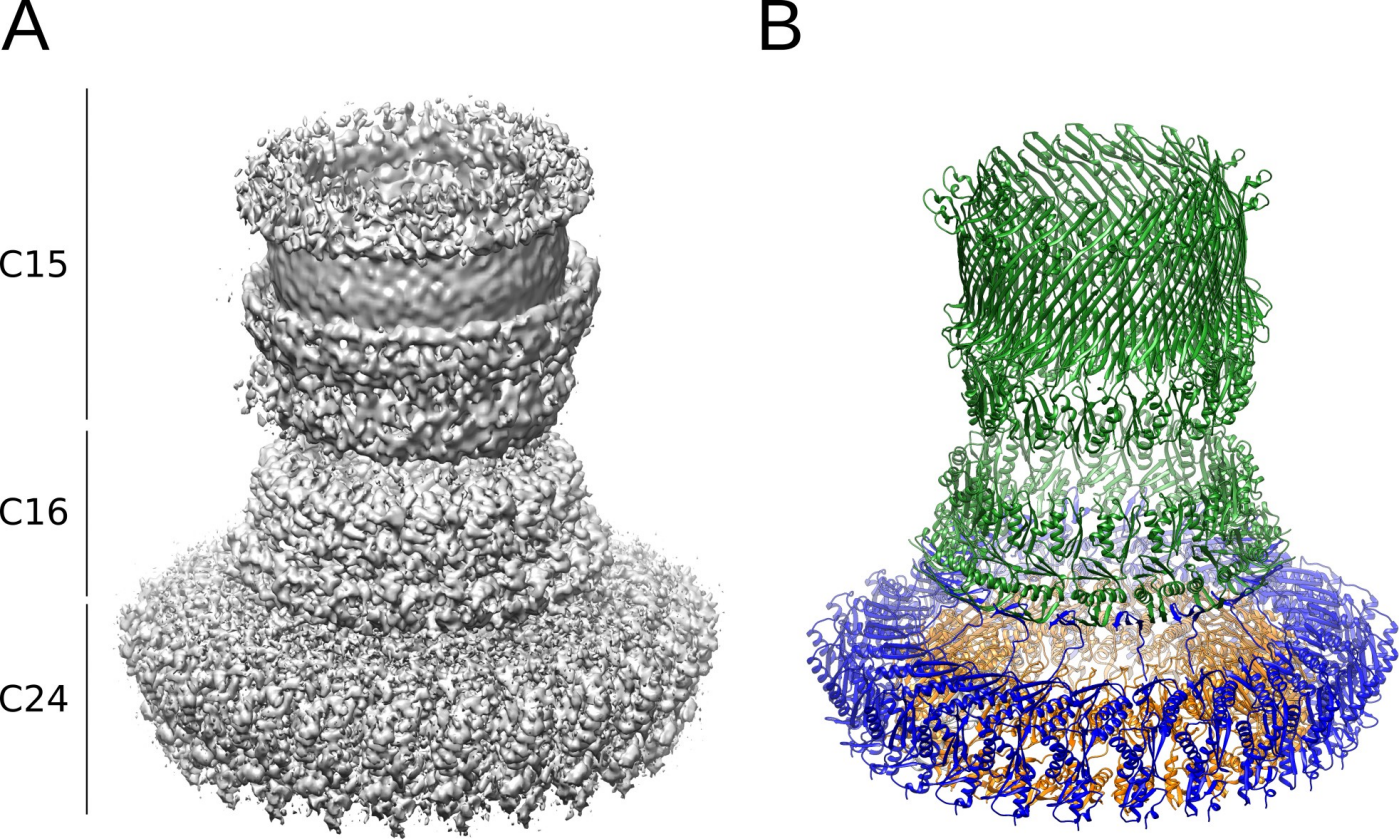
Architecture of the outer regions of the basal body. (**A**) Composite map of the highest-resolution maps obtained for the outer regions of the needle complex (left panel), including the focused IM ring map with C24 symmetry, the connector map from the focused IM-connector reconstruction with C8 symmetry and the OM ring map from C1 reconstruction. The cyclic symmetry of the three regions is indicated on the left. (**B**) Atomic models of the corresponding needle complex proteins built using maps depicted in (A): MxiG (blue), MxiJ (orange) and MxiD (green).

Consequently, we performed focused refinements for different regions of the needle complex to exploit the symmetry and obtain higher resolution maps (Table 1 and Fig 2). We applied C24 symmetry to the IM ring, consisting of the proteins MxiG and MxiJ, Using this strategy, we obtained a map at 3.6 Å overall resolution (S2C Fig and S3C Fig), which allowed manual building and refinement of an atomic model. To improve the connector map and resolve the interface between IM ring and connector, we performed a focused refinement of these regions imposing the common C8 symmetry. The resulting map with overall resolution 3.9 Å (S2B Fig) attains in the connector region local resolution in the range of 3.7 to 4.2 Å (S3B Fig) and shows the density of the backbone trace and bulky side chains of MxiD.

### The IM ring structure

**Fig 3:**
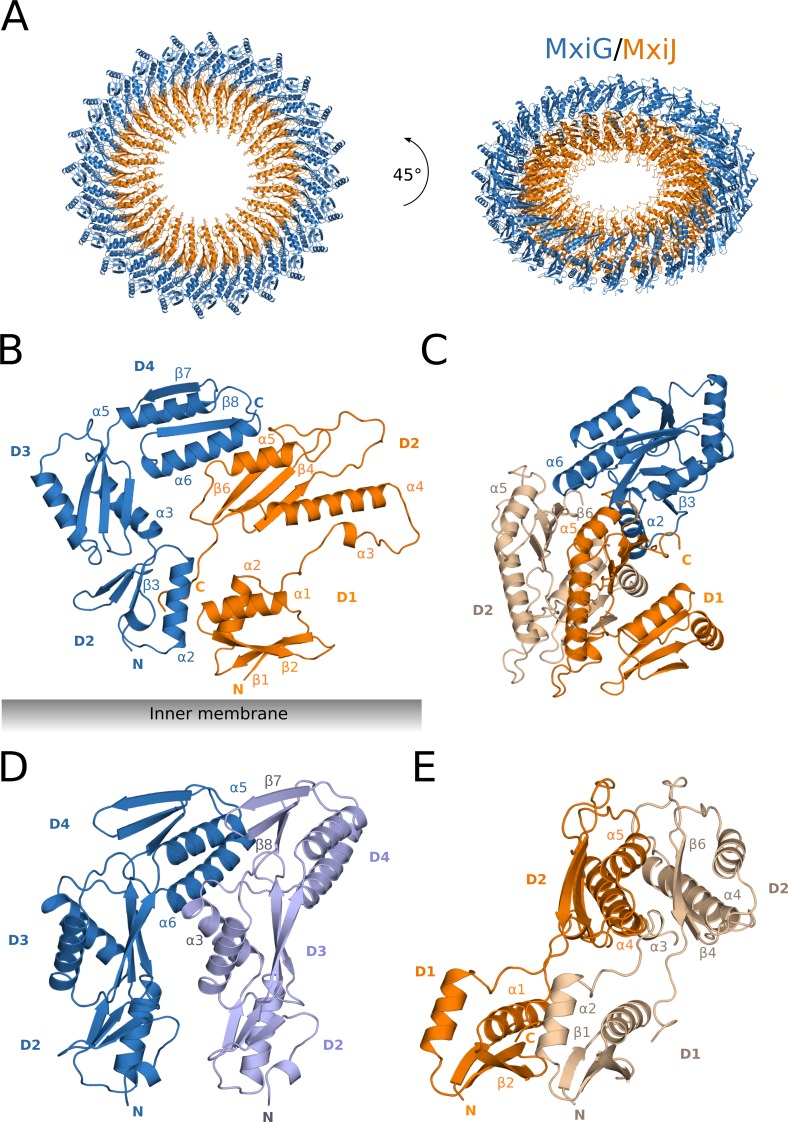
Structure of the periplasmic IM ring. The periplasmic IM ring model in cartoon representation (MxiG subunits in blue and violet, MxiJ subunits in orange and beige). (**A**) Top view (left panel) and tilted view (right panel) of the periplasmic IM ring composed by 24 copies of MxiG and MxiJ subunits forming two concentric rings. The inner membrane is located below the ring. (**B**) Side view of one MxiG and one MxiJ neighboring subunits. Only the periplasmic domains are shown and labeled. Secondary structure elements involved in IM ring interactions are also labeled. The domains adopt RBM αββαβ topology, except the D1 domain of MxiJ that has inverted RBM βαββα topology. (**C-E**) Interacting subunits of the IM periplasmic ring: one MxiG between two MxiJ subunits (C), a couple of neighboring MxiG (D) and MxiJ subunits (E). Secondary structure elements involved in IM ring interactions are labeled.

The periplasmic IM ring map shows clear backbone and side chain densities that allowed us to build and refine the first high-resolution experimental structures of the periplasmic domains of the IM proteins MxiG and MxiJ (Fig 3 and S4 Fig). Local resolution analysis indicates that the core of the periplasmic IM ring is resolved up to about 3.2 Å (S3C Fig). However, the density map for the cytoplasmic MxiG domain [24] is of lower quality and shows no structural features that allows the fitting of an atomic model. We suppose that this density comprises disordered detergent molecules and membrane lipids, together with the cytoplasmic domain of MxiG. As previously suggested [25], needle complex isolation solubilizes the bacterial membrane and leads to a closer localization of the cytoplasmic MxiG domain to the periplasmic IM ring,

The periplasmic IM ring is composed of 24 copies of the inner membrane proteins MxiG and MxiJ forming two concentric rings (Fig 3A). MxiJ forms the inner ring around the export apparatus structure, while MxiG assembles into the outer ring oriented towards the periplasmic space.

The MxiG N-terminal cytoplasmic domain (residues M1 to H125) is connected by a predicted transmembrane helix from residue S126 to L143 to the periplasmic domains D2 (E152 to L200), D3 (V201 to L272), D4 (S273 to I340) [24,26] and a final C-terminal domain (D341 to K371) which links the IM ring with the connector. MxiJ is composed of two domains, D1 (R21 to D77) and D2 (S97 to V191), separated by a nine amino acid long linker region (Fig 3B). A palmitoylation site at cysteine 18 and a C-terminal transmembrane helix anchor MxiJ to the inner membrane [27,28].

The domains D2 to D4 of MxiG and both MxiJ domains share a similar fold known as the ring-building motif (RBM), where two α-helices fold against a mixed three-stranded β-sheet [26]. The MxiG domains and the MxiJ D2 domain adopt an αββαβ topology, while the MxiJ D1 domain retains an inverted topology βαββα (Fig 3B).

Several homo- and hetero-interactions between MxiG and MxiJ stabilize the IM ring structure. Each protein molecule in the ring interacts with two identical subunits and two of the other type. The most extensive interface is provided by MxiJ dimerization with a surface area of ~2450 Å^2^, which corresponds to 20% of the monomer surface, whereas MxiG subunits share a relatively small homo-interface area of ~875 Å^2^ (8% of the monomer surface). The two MxiG-MxiJ interfaces have area of ~800 Å^2^ and ~650 Å^2^.

In MxiG, the D2 and D4 domains are mainly responsible for hetero-interactions. The helix α2 and the strand β3 of D2 are in contact with the C-terminal stretch (P188 –V197) of one MxiJ subunit and with the loop connecting the helix α1 and the strand β2 of the adjacent MxiJ subunit (Fig 3B and 3C and S5A and S5B Fig). In D4, the α6 helix of MxiG is located in the cleft formed by two α5 helices and a β6 strand of adjacent MxiJ subunits (Fig 3C). Notably, residue D311 at the N-terminal end of the α6 helix bridges two MxiJ subunits through the side chains of K167 and R168 of α5 helix and the backbone of V184 in the β6 strand (S5A Fig). The structural alignment shows that these residues of both proteins are mostly conserved among the SPI-1 family members (S6 Fig). No needle complexes could be isolated from mutants bearing an MxiG D311K, thus D311 appears to be essential for basal body formation. Furthermore, the ability to secrete effectors is abrogated (S7A Fig), as also reported for *Salmonella* [29,30].

MxiG homo-interfaces mostly involve the D3 and D4 domains, as a cleft separates the domain D2 of adjacent molecules, although long side chains can bridge the gap. For instance, K160 is hydrogen-bonded to Y172 of the neighboring MxiG subunit. In domain D4, the C-terminal end of the helix α5 of one MxiG subunit is packed against the sheet β7-β9 of the neighboring MxiG subunit. In addition, the C-termini of the helices α6 and α3 of two adjacent subunits are in contact (Fig 3D and S5C Fig).

MxiJ dimerization involves both domains. A network of hydrogen bonds, salt bridges and hydrophobic contacts connects helices α1, α2 and α4, most of the β-strands, the loop connecting α3 and α4, and the C-terminal stretch that folds back into the direction of the membrane (Fig 3E and S8 Fig). Several of the residues involved in homo-dimerization are conserved among orthologues (S6 Fig) and mutations of the residues E102, A104 or H130 abrogate secretion in the *Pseudomonas* T3SS [30]. Hydrophobic contacts involve mostly the D2 domain (S8C Fig). The highly conserved residue F90 is part of a hydrophobic patch at the MxiJ-MxiJ interface; mutation F90A leads to abrogated PrgK oligomerization in *Salmonella* [29]. The hydrophobic patch is in close proximity to a conserved loop (S6 Fig) flanked by prolines P91 and P99 that terminate the helices α3 and α4. This loop is located between the D1 and D2 domains of MxiJ and protrudes into the export cage, the region of space encompassed by the IM ring and the export apparatus. It reaches the central cup-like structure (S9A Fig) of the export apparatus, similarly to the tip of another loop that connects strands β4 and β5, although less conserved and highly charged (residues E136-N139). Interaction between this second loop and the *Salmonella* orthologue of SpaP has been observed also in crosslinking experiments [31].

### The periplasmic IM ring forms channels

**Fig 4:**
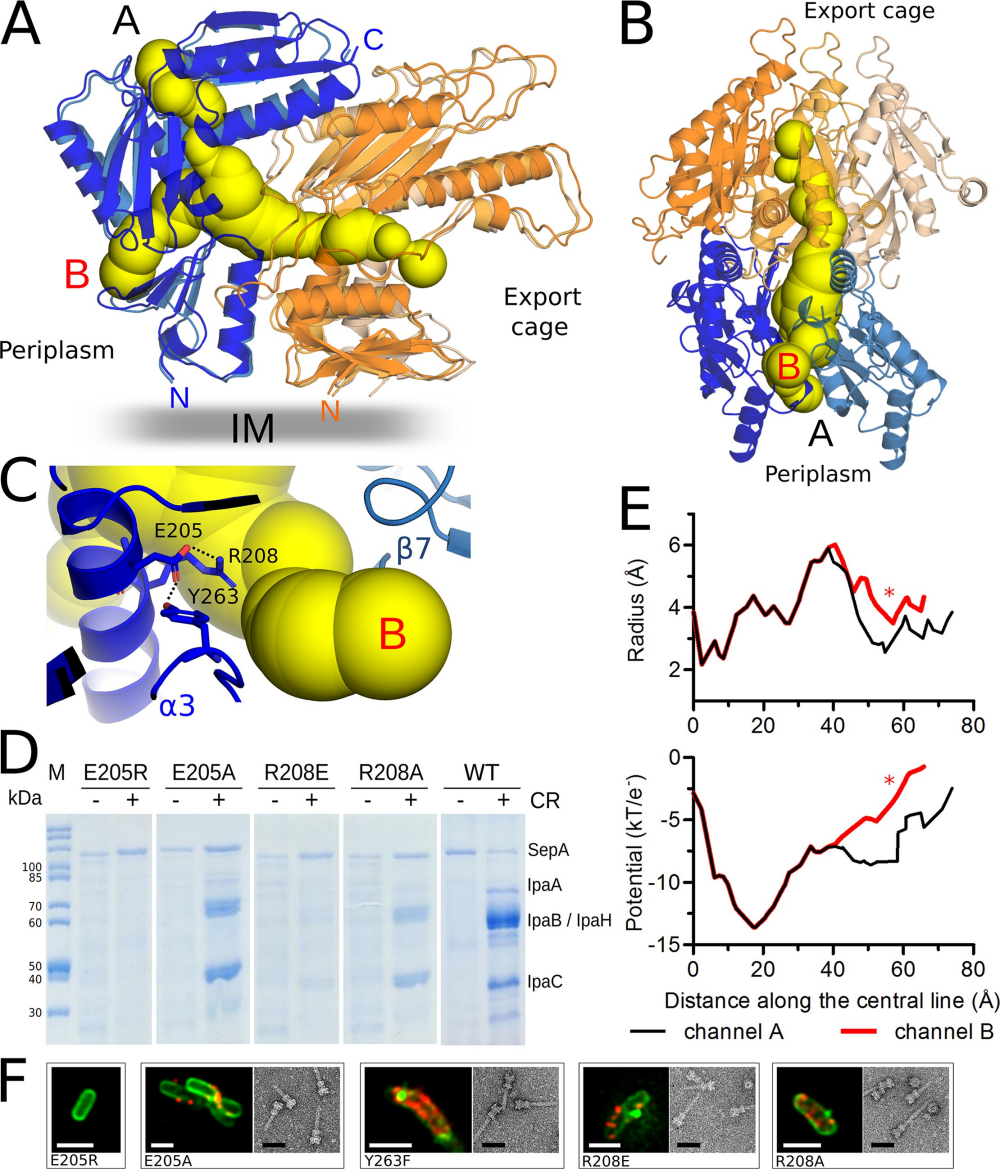
Channels inside the periplasmic IM ring of the *Shigella* needle complex. (**A**) Side view of the surface diagram of the channel connecting the export cage with the periplasm (yellow) at the interface between five IM ring subunits. Two MxiG (shades of blue) and three MxiJ (shades of orange) are represented as cartoons. The two channel exits towards the periplasm are labeled A and B. (**B**) Bottom radial view of the channel and subunits represented in panel (A). The channel runs between two MxiG (shades of blue) and three MxiJ (shades of orange) subunits. (**C**) Close-up of channel exit B indicates that the conserved MxiG residues E205, R208 and Y263 are located nearby a bottleneck. Hydrogen bonds involving their side chains are shown as dotted lines. (**D**) SDS-PAGE analysis of secreted proteins from wild-type (WT) and MxiG R208 and E205 *Shigella* mutant strains upon congo red induction. SepA serves as a T3S-independent loading control. IpaA, IpaB, IpaH and IpaC are T3SS effectors. Protein secretion was induced adding congo red (CR). (**E**) Channel radii and electrostatic potential profiles of the channels A and B plotted against the distance along the central line, starting from the export cage towards the periplasm. The asterisk indicates the location of the MxiG residues E205, R208 and Y263 near the channel exit B. (**F**) Representative immunofluorescence images of *Shigella* mutant strains and TEM images of negatively stained isolated needle complexes. In the immunofluorescence images the bacterial membrane is stained in green, T3SS needles in red. All the mutants except E205R produce the needle complex, which is localized on the membrane and it was successfully isolated. The white scale bar corresponds to 2 μm, the black scale bar of the TEM images to 50 nm.

The IM ring shows solvent accessible channels, formed by two MxiG and three MxiJ subunits, connecting the periplasm with the export cage (Fig 4A and 4B and S10A Fig). Twenty-four radially arranged channels branch out along the subunit interfaces giving a total length of 65 to 75 Å. The channels open on the export cage side at the MxiJ/MxiJ interface, below the P91-P99 loop. There are two exits on the periplasmic surface located between domains D3 and D4 (exit A in Fig 4A and 4B) and domains D2 and D3 (exit B in Fig 4A and 4B) of two MxiG subunits, respectively. The electrostatic potential along the central line of the channel is negative (Fig 4E). The channel has bottlenecks with ~ 2.2 Å radii near the export cage exit and ~3.0 or ~3.5 Å near the periplasmic exits A and B, respectively (Fig 4E). The conserved residues E205 and R208, part of the helix α3, and Y263 are located nearby the constriction close to exit B (Fig 4C, S6 Fig). The E205 side chain is hydrogen bonded with Y263 and R208. Single charge inversion of either E205 or R208 abrogates secretion, while single charge removal by mutation to alanine does not abolish secretion or needle complex formation (Fig 4D and 4F). Interestingly, R208E can form intact needle complexes, while needle assembly is impaired in the E205R mutant (Fig 4F) Y263, located in a loop at the D2-D3 interface is hydrogen bonded with E205 via the side chain hydroxyl group. Removing the hydroxyl group from Y263 (Y263F) reduces effector secretion, though the structural integrity of the needle complex is maintained (Fig 4F, S7C Fig). The single point mutation E205R and the double mutant Y263F_E205R show impaired effector secretion as well, but no intact needle complexes could be isolated, though both mutant proteins are integrated in the bacterial membrane (S7C and S7D Fig). Therefore we conclude that local changes in the electrostatic potential along the channel, or conformational changes triggered by the repulsion of sidechains, could be critical for the T3SS function. Further, a circular channel, formed between the α3 and α6 helices of MxiG and the loop connecting β5 and α5 of MxiJ, interconnects the 24 radial channels (S10A Fig). Though many residues lining the channels are not conserved, a tubular network similar in position, dimensions and electrostatic potential can be found in the IM ring of the *Salmonella* basal body (S10B and S10C Fig).

### The connector structure and the interface with the periplasmic IM ring

**Fig 5:**
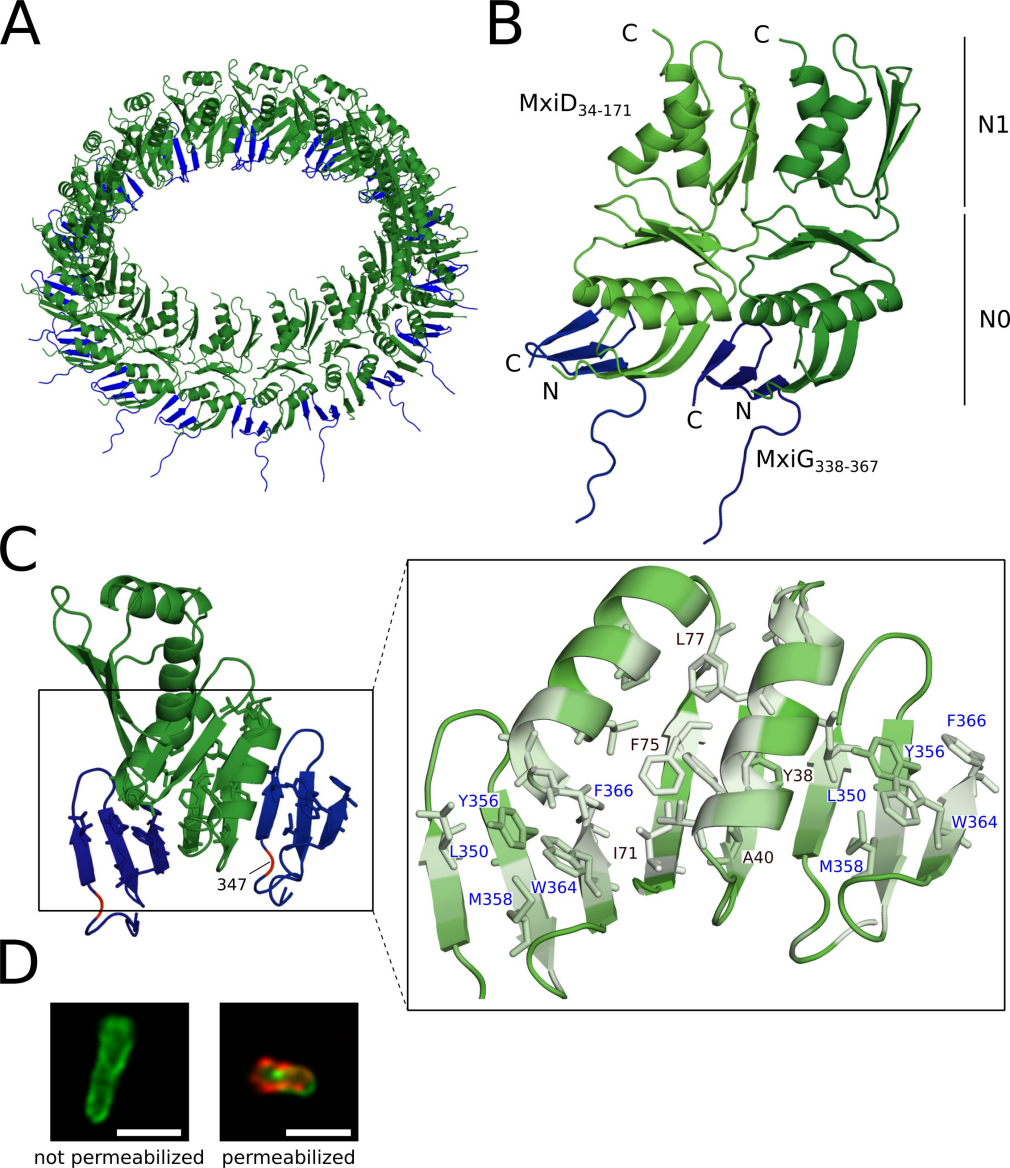
Structure of the connector and its interface with the IM ring. **(A)** Tilted top view of the connector composed of the N-terminal N0 and N1 domains of MxiD (green) and the C-terminal domain of MxiG (blue) in cartoon representation. The ring is formed by 16 subunits of each protein. **(B)** Close-up of two neighboring couples of MxiD and MxiG subunits. The MxiG subunits interact with the N-terminus of MxiD via β-sheet augmentation, forming a continuous circular β-sheet at the connector base. **(C)** N0 and N1 domains of MxiD between the C-terminal domain two MxiG subunits (left panel); residues represented as sticks are involved in intermolecular hydrophobic interactions, the position of the amino acid 347 in MxiG is indicated in red. Detailed view of the MxiG-MxiD hydrophobic interface colored according to the Eisenberg scale (white being the most hydrophobic) (right panel); black-labeled residues are at the interface and belong to MxiD (Y38, A40, I71, F75, L77 and A36, which is not visible in this view), MxiG residues are labeled in blue (L350, Y356, M358, W364 and F366). (**D**) Representative images of immunofluorescence stained *Shigella* M90T ΔMxiG complemented with truncated Strep-MxiG comprising residues 1 to 346 (MxiG-347-Stop). Green, lipophilic membrane stain; red, anti-Strep-tag antibody. *strep-mxiH* and *strep-mxiG* genes are expressed from a plasmid in non-permeabilized bacteria while permeabilized bacteria expressed only *strep-mxiG*. No needle complex is visible on the surface of the bacteria, while the MxiG mutant is localized in the bacterial membrane. The scale bar corresponds to 2 μm.

Homo-oligomerization of the secretin MxiD leads to the formation of the connector and OM ring regions [5]. The former includes sixteen copies of the N-terminal domains N0 (residues N23-E109; the first 22 residues belongs to a cleaved signal peptide) and N1 (residues L110-K171) and appears to be disjointed from the IM ring by a gap about 15–20 Å wide (Fig 1A). The focused C8 map (Fig 2A) allowed us to model and refine the entire connector region of the *Shigella* needle complex (Fig 5A and S11A Fig). The interaction with the IM ring is established exclusively through the N0 domain of MxiD (Fig 5B). Matching of the different symmetries of the IM ring (C24) and the connector (C16) is achieved through the C-terminal domain of MxiG, which can adopt different conformations. The MxiG ring can be seen as an assembly of eight triplets; the C-terminus of two subunits of each triplet folds into a three-stranded twisted anti-parallel β-sheet (residues 348–366) that adds to two β-strands of the MxiD N0 domain via a β-sheet augmentation [32] (Fig 5B, S11B Fig). The MxiD β-sheet is bent almost in the middle and thus divided in two halves; one half interacts with the preceding MxiG strands, while the second half is bound to the following MxiG β-sheet, which adapts well to the bent MxiD strands thanks to its twist (Fig 5B). Therefore, the base of the connector is a continuous circular β-sheet. An amino acid stretch (residues 341–345) covers the ~15 Å distance between the IM ring and the connector, linking the previous MxiG domain with the C-terminal β-sheet at the connector base (S11C Fig). The conformation of this stretch is different in the two subunits of the triplet to allow adaptation of the C24 architecture of the IM ring with the C16 of the connector (Fig 5B), while the conformation of the β-sheets shows no major differences. One MxiG subunit of the triplet interfaces MxiD with a surface area of ~500 Å^2^, while for the other it is ~430 Å^2^. Insertion of a stop codon at position 347 shows that the C-terminal MxiG β-sheets is essential for needle assembly and effector secretion (Fig 5D and S7C Fig). Using fluorescent imaging, no needles could be detected on the surface of *mxiG* knock-out bacteria expressing the *mxiG-347-stop* mutant gene, though the protein was integrated in the bacterial membrane (Fig 5D). Attempts to isolate the basal body from bacteria producing strep-tagged MxiG-347-stop were unsuccessful, suggesting that the basal body is not assembled.

Our density map also reveals that the C-terminus of the third MxiG subunit of the triplet is mostly located in the space between the connector and the IM ring instead of forming a β-sheet in the connector (S11C Fig). However, the quality of the corresponding density is not good enough to allow reliable *de novo* modeling of its atomic structure.

Two amphipathic helices of the N0 domain lie on top of the β-sheet with the hydrophobic side facing each other and the β-sheet (Fig 5C). Here, most of the interacting residues are conserved or substituted with similar hydrophobic amino acids among species (S6 Fig and S12 Fig). These hydrophobic interactions are continued along the entire ring and, together with the backbone hydrogen bonds of the augmented β-sheet, may substantially contribute to stabilize the complex between the MxiG and MxiD oligomers. A three-stranded twisted and mostly hydrophobic β-sheet completes the N0 domain on top of the two helices. The last two strands and the turn connecting them are facing the N1 domains of the same subunit (surface area ~470 Å^2^). The N0-N1 interface is stabilized by three hydrogen bonds and hydrophobic interactions involving several conserved residues (S13 Fig). Remarkably, the highly conserved aromatic side chains of Y96 and Y105 are also forming hydrogen bonds.

The N1 domain adopts the inverted βαββα RBM architecture. The residues in contact with N0 of the same subunit are mostly located in loops connecting secondary structural elements, which in turn are involved in intermolecular interactions with the adjacent subunits. Altogether, the N1 domains form a ring where a three-stranded β-sheet alternates with two α-helices. The surface area of the N1-N1 interface (~ 465 Å^2^) is moderately hydrophobic (S14B Fig) supporting five intermolecular hydrogen bonds (S14A Fig). A second interface between two adjacent MxiD subunits enforces the stability of the connector ring. This interface is formed by the N1 β-sheet and the N0 three-stranded β-sheet together with the loops connecting its strands (surface area ~305 Å^2^) and is stabilized by five hydrogen bonds (S14A Fig). In particular, the conserved residues D98 and N100, belonging to the turn between the last β-strands of N0, are involved in multiple hydrogen bonds. Residues of this turn participate in polar N0-N1 interactions also in the *Salmonella* orthologue InvG [33] (S14C Fig).

The N0-N0 interaction completes the interfaces between adjacent MxiD subunits. It involves the end of the two helices, the loops connecting them with the three-stranded β-sheet and the loop linking N0 and N1. The small interface (~280 Å^2^) includes a single intermolecular hydrogen bond between S107 and I60.

### Architecture of the OM ring

**Fig 6:**
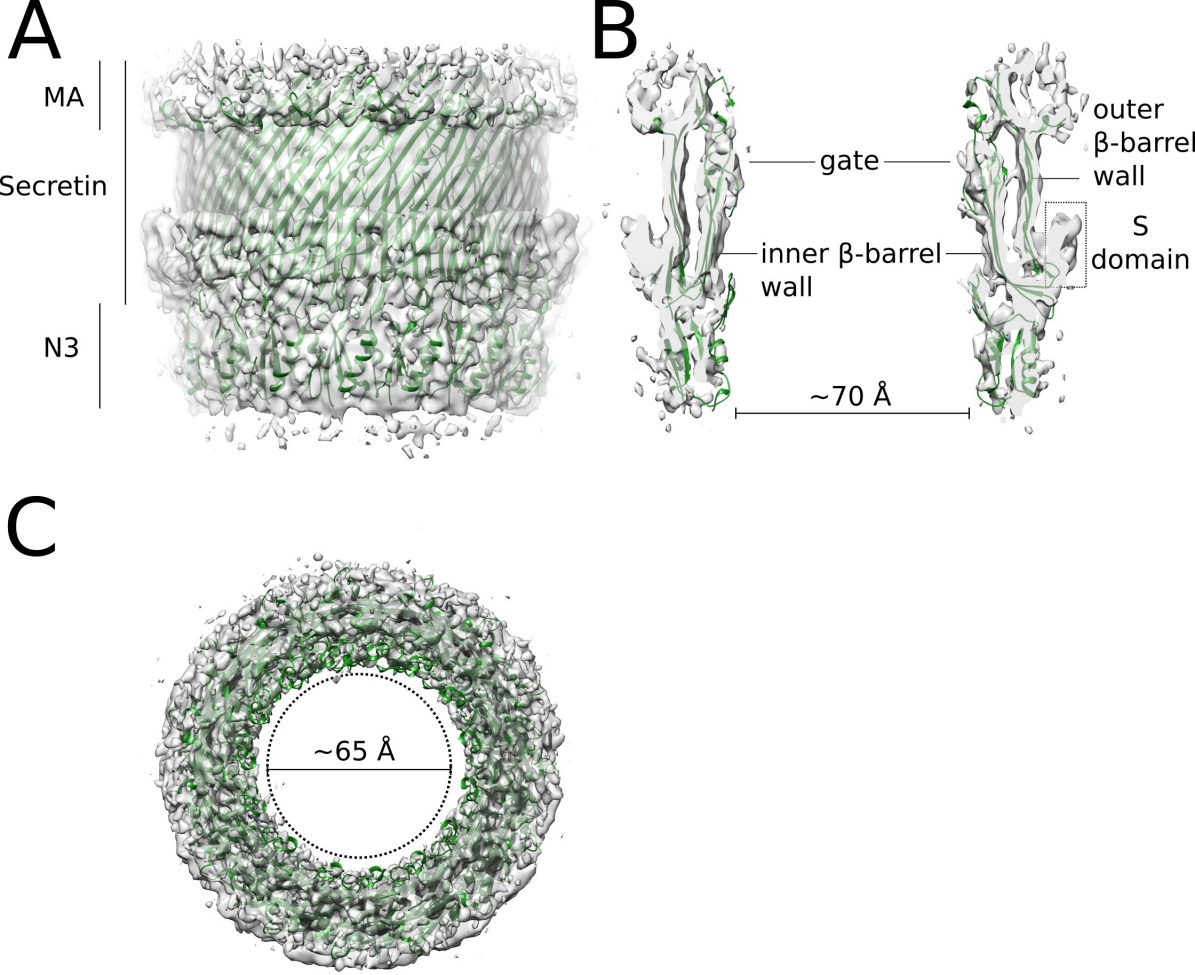
Architecture of the OM ring. **(A)** Cartoon representation of the vertical cutaway of the MxiD pore in the C1 map. The subunits form a barrel shaped ring system that is anchored to the outer membrane. The OM ring is composed of the RBM N3 domain, the central secretin domain and its membrane associated (MA) subdomain. **(B)** A vertical slice of the OM ring in the C1 map. The inner and outer β-barrel walls are indicated. The MA subdomain, the periplasmic gate and the N3 domains form constrictions with diameter ~70 Å. The C-terminal S domain of MxiD is likely located on the outer side of the β-barrel in the indicated region. **(C)** Top view of the OM ring cartoon fitted into the C1 map. The dotted circle represents the outer diameter of the needle filament, which passes through the OM pore.

The other domains of the secretin MxiD form the OM ring of the *Shigella* needle complex. Similarly to the *Salmonella* orthologue InvG [34], the N1 domain is followed by the N3 domain (residues D179-H300), the secretin domain (residues I301-I520), and finally the small C-terminal S domain (residues K521-Y566) (Fig 6A). The upper part of the secretin domain that is embedded in the outer membrane can be identified as the membrane associated (MA) subdomain. As found in our *Shigella* C1 map and in agreement with a recent report for the *Salmonella* needle complex [23], the OM ring and the connector might adopt different symmetries, with the former comprising 15 instead of 16 subunits (Fig 1B and 1C). We built a model of the *Shigella* OM ring fitting homology models of the N3 and secretin domains, based on the structure of the InvG open conformation [33], into the unsymmetrized C1 map. Due to the low conservation of sequence and structure among secretins [34,35] (S12 Fig), we did not include the S domain.

We fitted the N3 and secretin domains independently in our C1 map as rigid bodies. The fifteen copies of each domain fill the density without major clashes (Figs 6 and 2B) forming a pore with minimal diameter ~70 Å, wide enough to accommodate the T3SS needle (Fig 6C, S15A Fig). The secretin domain forms a massive double-walled antiparallel β-barrel with the same diameter as in the *Salmonella* orthologue. The upper ends of the four β-strands of the inner wall make two extended loops termed the periplasmic gate. One of these loops, which forms a twisted β-hairpin, functions as the gate that closes the pore in absence of the needle. The β-barrel fits well the map and it shows the distinct locations of the inner and outer walls (Fig 6B). The lower half, bent towards the outside, and likely the S domain form the circular rim visible in the map. At the distal side of the outer β-barrel wall, the MA subdomain forms a circular groove with a hydrophobic surface (S15C Fig) that interfaces with the outer membrane lipids.

The proximal edge of the β-barrel is in contact with the ring formed by the N3 domain (Fig 6A). This domain shares the βαββα RBM architecture with the N1 domain and has two additional insertions. One of them is a β-hairpin (residues S194-G209) that supports the hairpin gate in the closed conformation, and makes an extension of the inner β-barrel wall in the open conformation. The other insertion (residues N231-S258) is not conserved (S12 Fig) and only partially ordered in InvG, thus we excluded it from the model. The diameter of the N3 ring is similar to *Salmonella* and like in *Salmonella* the orientation of N3 is the opposite as the N1 domain. The N3 model reveals the hydrophobic nature of the β-sheet and the amphipathicity of the two α-helices, suggesting that these secondary structure elements are strong contributors to the formation and stability of the secretin oligomer. The relatively big extension of the surface area of the N3-N3 interface (~710 Å^2^) supports the relevance of N3 domain in driving the oligomerization process [34].

### Architecture of the export apparatus and the inner rod

The C1 map of the needle complex shows distinct tubular densities at the resolution of 5–6 Å in both the export apparatus and the inner rod and needle (S3A Fig), suggesting the presence of α-helices. We built and fit homology models to reveal the arrangement of the protein components of the inner regions of the basal body.

**Fig 7:**
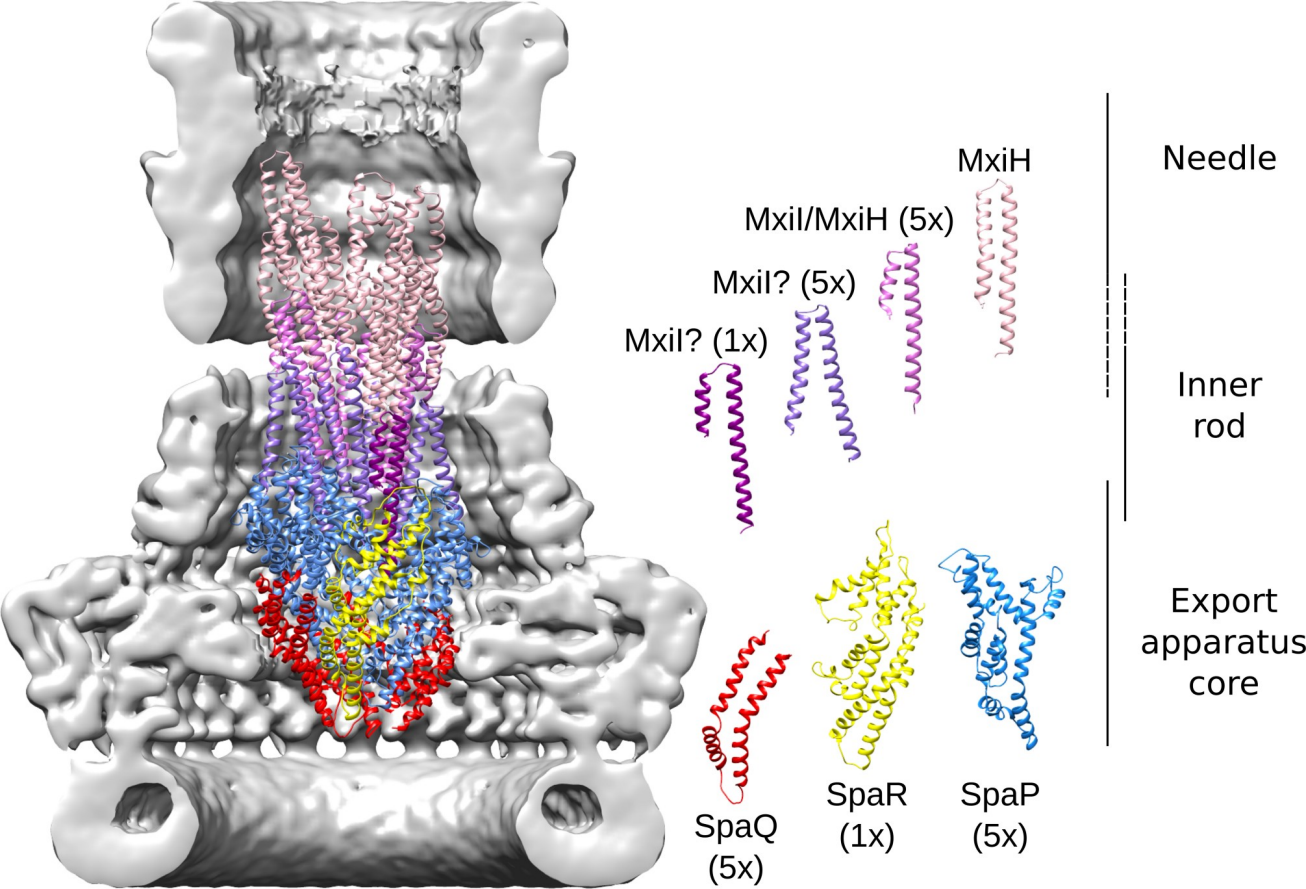
Architecture of the export apparatus core, inner rod and start of the needle. Cartoon representation of the subunits forming the export apparatus core, the inner rod and the start of the needle filament, in a vertical section of the outer needle complex. The individual protein components are depicted on the right side. Five SpaP, five SpaQ and one SpaR subunits, arranged in a helical fashion, form the export apparatus core. The subunits connecting the export apparatus with the needle were modeled as polyalanine peptides and adopt three different conformations (see the main text discussion about their possible identity). They share a helix-turn-helix fold and continue the export apparatus helix forming two turns. The upper turns are likely composed of needle subunits MxiH.

We used the recently published cryo-EM structure of the isolated export apparatus core from the *Salmonella* flagellar system [31] as templates for modeling the structures of the *Shigella* proteins SpaP, SpaQ and SpaR. These proteins are mostly composed of helices, which allow the fit into the low-resolution map. Random search followed by rigid body and flexible fitting allowed us to place five subunits of SpaP and SpaQ, respectively, and one of SpaR into our C1 map (Fig 7 and S16 Fig). The turn region of SpaQ, the smallest of the three proteins, which forms a kinked helix-turn-helix motif, is increasingly poorly defined, from top to bottom, probably due to its flexibility, and therefore not modeled in three subunits.

As in *Salmonella*, the subunits of the *Shigella* export apparatus adopt a conical shape with a cavity inside (Fig 7, S9 Fig). Five helically arranged SpaP-SpaQ dimers build a turn terminated on the top by one SpaR, which has been suggested to resemble the fusion between SpaP and SpaQ [31]. SpaQ subunits are located on the outer surface of the proximal part of the complex, while the bigger SpaP protein forms the inner and distal regions. Analysis of the helical parameters indicates a helix pitch of 19.8 Å and 5.8 subunits per turn, similar to the parameters reported for the *Salmonella* flagellar complex [31] and for the *Shigella* needle filament [6,36].

The export apparatus also interacts with two MxiJ loops protruding from the IM ring (S9A Fig). The atomic model shows that the lower loop, which is more hydrophobic, interacts mainly with the surface SpaQ subunits. The second and more hydrophilic loop (S6 Fig) is in contact with SpaP, which has a more polar surface than SpaQ.

The so-called ‘gasket’ SpaP loops, which have four methionine residues in the *Shigella* export apparatus, close the bottom opening to the positively charged inner cavity (S9B Fig). The open side of the cavity faces the inner rod and forms a continuous channel that extends into the needle filament. In the isolated *Salmonella* structure, however, the N-terminal helix of the distal SpaP subunit obstructs the connection with the inner rod, likely because it is not stabilized by interactions with the neighbouring inner rod subunits (S17A Fig).

We built a model for the inner rod fitting in our map a few turns of the published *Shigella* needle structure obtained by solid state NMR and EM [37]. The rod protein MxiI has similar size and predicted helix-turn-helix topology as the needle protein MxiH (S17B Fig). The fit of the needle structure shows that all the tubular densities in the inner rod region may be occupied by subunits having this topology (Fig 7 and S16 Fig). The inner rod has the same right-handed helical architecture as the needle with eleven subunits per two turns. However, we observed differences in the subunit conformations after flexible fitting. No clear density is visible for the first half of the N-terminal helix of the most proximal subunit, located directly above SpaR, while both helices are visible and diverging from each other in the following five subunits that complete the first turn. In the five subunits of the second turn, helices are also not parallel but with a shorter density for the N-terminal helix. The subunits composing the following turns appear to have a remarkably similar conformation to that of the isolated MxiH needle, besides forming an identical helical assembly. Except for the first 10 residues, not visible in our map and modeled as a non-helical loop in the isolated needle structure, MxiH superposes well with the fitted subunit with Cα root mean squared deviation (RMSD) of 0.68 Å. Thus, we modeled these protein subunits as MxiH. On the other hand, since we are uncertain about the identity of the subunits forming the first two turns, we modeled them as alanine polypeptides (see the discussion below).

## Discussion

Identification and analysis of the conserved and variable features of T3SS from different bacterial species is essential to understand the molecular mechanisms of effector protein secretion and the infection processes that depend on it. The shape and overall dimensions of the *Shigella* needle complex and the 24-fold symmetry of its IM ring is in agreement with earlier studies in *Shigella* [38], *Salmonella* SPI-1 system [21,33], *Yersinia* [39] and *E*. *coli* [40]. The suggested 12-fold symmetry for the *Shigella* IM ring however, could not be confirmed by our analysis, which might be due to the negative staining or the limited resolution of the previous study [41]

The RBM architecture of the domains of MxiG and MxiJ subunits is conserved in the *Salmonella* orthologues. Individual domains superpose with Cα RMSD better than 1.5 Å (S18 Fig). The RBM domain is also found in other secretion system components such as InvA from the *Salmonella* export apparatus [42,43] and various secretins including MxiD in *Shigella* [13], underlining its importance for the ring assembly in bacterial membrane complexes. Superposition of the MxiD N0 and N1 domains with the corresponding domains of InvG gives somewhat bigger RMSD ~1.6 and ~2.5 Å, respectively, possibly because of the 15-fold symmetry assumed upon refinement [33].

How the different symmetries of the needle complex components are compatible with one another is still an open question. We found that the C-terminal domain of MxiG adopts multiple conformations to provide a link with the connector. The outer IM periplasmic ring arranges in eight triplets of MxiG subunits to match the 16-fold symmetry of the connector. Two MxiG subunits of each triplet bind the N0 domain of MxiD via a β-sheet augmentation, a common mechanism for protein-protein interaction found in cell-signaling pathways, ion channels, receptor activation [32,44] and type II bacterial secretion [45]. The IM ring-connector interaction, mediated by the C-terminal domain of MxiG, is critical for the assembly of the T3SS basal body and observed for the first time in this study. In *Salmonella*, deletion of the last four C-terminal amino acids of PrgH together with high pH treatment destabilizes the needle complex and disassembles it into IM and OM rings [7]. Along these lines, the interaction between the C-terminal domain of PrgH, *Salmonella* orthologue of MxiG, and the N-terminal domain of InvG was suggested by cross-linking experiments [46] and the deletion of the C-terminal domain of the *Yersinia* YscD abolishes binding with YscC, orthologues of MxiG and MxiD respectively [47].

Although it was not possible to determine precisely the conformation of the C-terminal domain of the third MxiG subunit, our map shows that this domain locates at the IM ring and connector interface in *Shigella*. It might also interact with the N-terminal residues of MxiD, not resolved in the map, or with additional periplasmic components lost during isolation of the needle complex.

The 2+1 organization of the C-terminal MxiG domain and the β-sheet augmentation explains how the 24-fold symmetry of the IM ring adapts to the 16-fold symmetry of the connector. Notably, the 16-fold symmetry of the *Shigella* connector differs from the 15-fold symmetry reported in early reconstructions of the *Salmonella* T3SS basal body [21,33,34] and of the type II secretion system secretins of *Vibrio cholerae* and *Escherichia Coli* K12 [35]. However, the latter system can also adopt 16 fold symmetry [35] and a recent study provides evidence that even the *Salmonella* T3SS connector is formed by 16 subunits [23].

In structures obtained from isolated secretin oligomers the connector N0 domain appears to have a high degree of flexibility [34,35]. The suggested stabilizing effect of the IM ring is supported by our analysis of the *Shigella* needle complex, where the connector region is well defined and the MxiD-MxiG interface has large extension (~430–500 Å^2^).

Intriguingly, the unsymmetrized map of the *Shigella* needle complex shows C16 and C15 symmetries for the connector (N-terminal domains N0 and N1 of MxiD) and the OM ring (C-terminal domains N3, secretin and S of MxiD), respectively. Likely, this asymmetry is responsible for the tilt between connector and OM ring observed in the reconstruction (Fig 1A, S3A Fig). The role and location of the C-terminal domains of the 16^th^ MxiD subunit is unclear, as no density could be assigned to it. The assembly of the mixed C15-C16 oligomer needs to be further investigated. It could be initially composed by 16 subunits, and the C-terminal domains of the 16^th^ subunit are expelled at a later stage. Alternatively, the secretin pore assembly can be explained by a common mechanism of oligomerization of 15 subunits, and the 16^th^ N-terminal domains are incorporated when the oligomer binds the IM ring, to adapt to its C24 symmetry [23]. This hypothesis is supported by the pentadecameric arrangement observed in isolated secretin oligomers [34,35] and by the flexibility of the N-terminal domains observed in these structures, which might allow the insertion of the 16^th^ subunit upon IM-ring binding.

Interestingly, the inner surface of the pore and the outer surface of the needle are mostly negatively charged under physiological conditions (S15A and S15B Fig). The electrostatic repulsion may help in keeping the needle in the correct position at the center of the secretin pore. In addition, the repulsion may promote the pore opening and enhance the steric pressure of the growing needle on the periplasmic gate [33].

A proton motive force (PMF) is present across the bacterial inner membrane [18,19,48,49]. The PMF, which is an electrochemical gradient established by the pH-and charge differences between the periplasm and the cytoplasm, has been shown to be indispensable for substrate transport through T3SSs in *Pseudomonas* and *Yersinia* [16,17] and for flagella rotation in *Salmonella* [18,48,50,51]. It has been suggested that protons are transported via FlhA in the *Salmonella* flagellar system to the associated cytoplasmic ATPase complex [52,53]. The *Shigella* T3SS homologue MxiA, which might be lost during the isolation of the needle complex since it is not visible in the map, is a transmembrane protein associated to the export apparatus [10,13,54]. Its N-terminal domain is expected to be located in the export cage, in close proximity to the opening of the IM ring channels. We speculate that the polar channels found in the IM periplasmic ring are water-filled and thus could provide a pathway for proton flux. Since proton transport through water channels requires charge transmission between adjacent water molecules [55], it is plausible that local conformational changes of the IM ring subunits could interfere with this process and the overall function of the T3SS. In particular, the residues R208 and Y263 stabilize the charged E205 close to a channel bottleneck; their mutation does not prevent needle assembly but abrogates secretion, possibly because of changes in the structure or charge distribution along the channel. Additionally, interaction with regulatory proteins might also lead to conformational rearrangement of the IM ring that could influence proton flux and subsequent effector secretion.

The IM ring and the export apparatus interact through a structurally conserved loop comprising residues P91 to P99 in MxiJ. The hydrophobic residues of this loop might promote the close contact between these two needle complex components despite their different symmetries and allowing some rotational freedom relative to each other for functionality [56], as a hydrophobic interface might require the lowest activation energy compared to other molecular interactions.

We found evidence in our map for a 5:5:1 stoichiometry of the export apparatus proteins SpaP:SpaQ:SpaR of the *Shigella* T3SS. This molecular arrangement differs from the 5:4:1 stoichiometry found in the reconstruction of the isolated export apparatus of *Shigella* [54] and *Salmonella*. The difference could be consequence of the isolation process of the complex or its heterologous overexpression. Native MS reveals the presence of the 5:5:1 complex when purified with a mild detergent in *Pseudomonas savastatoi* [31]. However, *Salmonella* cross-linking data suggest that the additional subunit SpaS is also bound close to the location that we assigned to the 5^th^ SpaQ [31]. Therefore, we cannot exclude the presence of a different subunit.

Comparison of our structure with the isolated export apparatus showed different conformation at the inner rod interface, where the N-terminal helix of a SpaP subunit occludes the opening in the isolated export apparatus, while it is bent and moved aside in the needle complex. The helix movement provides additional space for the passage of transported molecules (S17A Fig). Our C1 map shows the transition between the export apparatus and the inner rod. We modeled the eleven subunits forming the first two turns as alanine polypeptide with helix-turn-helix conformation *In vivo* cross-linking studies in *Salmonella* have shown that PrgJ (MxiI orthologue, the inner rod subunit) interacts with SpaP and SpaR [11,57]. A stoichiometric analysis of the *Salmonella* needle complex revealed the presence of only few copies of PrgJ [58], suggesting a limited extension of the inner rod, and more recently the inner rod has been reported to be composed of only one turn of PrgJ molecules [23,57], which acts as an adapter between export apparatus and needle filament. Thus, it is likely that MxiI forms at least the first turn of the inner rod/needle, while the second turn could be formed either still by MxiI, or by the needle protomer MxiH adopting a different conformation than in the rest of the needle (Fig 7). The remarkable conformational similarity between the published MxiH structure [37] and the subunits forming the following turns strongly suggests that they are composed by the needle protomer. Interestingly, the secondary structure prediction of MxiI indicates the presence of two helices connected by a coiled region around the twentieth residue, which corresponds to the position where we observe the interruption of the N-terminal helix in six of the first eleven subunits (Fig 7 and S17B Fig). The first half of the N-terminal helix could be more flexible or even disordered, and thus not resolved in our map.

The structure of the *Shigella* needle complex described in this study contributes to a better understanding of the molecular mechanisms underlying protein secretion, with the ultimate aim of supporting the development of novel therapeutics to fight against multi-drug resistant Gram-negative bacteria that employ the T3SS.

## Materials and methods

### Bacterial strains and cell culture

The knockout *Shigella* mutant M90T *ΔIpaDΔMxiH* was generated by λ Red recombination [59]. M90T *ΔIpaDΔMxiH* was complemented with N-terminal Strep-tagged MxiH encoded on an anhydrotetracycline hydrochloride (AHT) inducible plasmid pASK-IBA5plus. M90T *ΔMxiG* was complemented with an N-terminal Strep-tagged MxiG encoded on an AHT inducible plasmid pASK-IBA5plus. For co-localization of the T3SS needle with the bacterial membrane, N-terminal Strep-tagged MxiH encoded on pASK-IBA3C was co-transformed with Strep-MxiG on pASK-IBA5plus in M90T *ΔMxiG*. *Shigella* were kept on Tryptic Soy Broth (TSB) agar plate supplemented with 0.01% (w/v) Congo red and antibiotics as selection markers. *E*.*coli* DH5α were kept on lysogeny broth (LB) agar plates with antibiotics as selection markers.

### Isolation of the needle complex

Bacteria were grown in TSB at 37°C, MxiH expression was induced by AHT to final concentration of 0.2 μg/ml and bacterial cells were harvested at OD_600_ 0.4–0.6. Cells were washed with phosphate buffered saline (PBS) and osmotically shocked by resuspension in buffer containing 18% (w/v) sucrose, 1 mg/ml Lysozyme, 100 mM Tris pH 8.0, 100 mM NaCl and 1.25 mM EDTA at 37°C, until vast majority of bacteria formed spheroplasts. Cell lysis was performed with 2% (v/v) Triton-X-100, in the presence of DNAse 1 and 10 mM MgSO_4_ and Complete Ultra Protease Inhibitor Cocktail (PIC; Roche) at room temperature for 15 min. Cell debris was removed by centrifugation in a JA25.50 rotor at 15000 rpm at 4°C for 20 min. The resulting supernatant was centrifuged at 40,000 rpm with rotor 45Ti at 4°C for 4h, the pellet was solubilized in 50 mMTris pH 8.0, 5 mM EDTA, 100 mM NaCl, 0.02% w/v n-Dodecyl β-D-Maltopyranoside containing PIC at 4°C overnight. Needle complexes were isolated using Strep-tactin sepharose resin (IBA Lifescience) and used immediately for further structural analysis.

### Cryo TEM data collection

400-mesh Quantifoil R2/1 holey grids with additional ~4–5 nm continuous thin carbon layer were incubated with 5 μl of sample and vitrified with FEI Vitrobot Mark IV. Different glow-discharging, incubation and blotting times were tested and the grids were inspected either at the electron microscope FEI Tecnai Spirit at the Max Planck Institute for Molecular Genetics (Berlin, Germany) or FEI Tecnai G2 F20 in CEITEC (Brno, Czech Republic). 5,238 micrographs were collected with a FEI Titan Krios microscope in CEITEC (Brno, Czech Republic) at 300 kV equipped with a FEI Falcon II detector from a grid vitrified after 15 sec glow-discharging, 5 min sample incubation on ice and 2 sec blotting time. Each micrograph is composed of 7 frames, with a total exposure time of 1.5 sec per micrograph and total dose of 25 e^-^/Å^2^. Pixel size was 1.38 Å, corresponding to a magnification of about 100,000 fold. Nominal underfocus value was set randomly in the interval 1.5–4 μm.

Negative-stained micrographs of needle complex from *Shigella* mutants have been collected with a FEI Talos L120C microscope at the cryo-EM facility of the Centre for Structural System Biology (Hamburg, Germany). Samples were stained with 1% uranyl acetate.

### Cryo TEM data processing

Individual frames were aligned with Motioncorr 2.1 [60] and the program xmipp_movie_optical_alignment from the software suite Xmipp 3.2 [61]. 197 micrographs were removed due to contamination or drifting effects based on examination of images and corresponding power spectra. Defocus values were estimated with CTFFIND 4.0.17 [62]. Relion 1.4 and 2.1 [63] was employed for subsequent data processing. About 1,400 particles were manually selected and subjected to 2D classification. 5 classes were selected as templates for autopicking. 171,833 particles were initially obtained and sorted according to similarity to the reference images, which allowed removal of 28,347 false particles. The remaining particles were subjected to three rounds of 2D classification. After each round, the particles belonging to noisy classes were removed. This process yielded a dataset with in total 104,272 particles, which were used for 3D classification including three classes and using as reference a needle complex reconstruction at about 25 Å resolution obtained from a previously collected dataset (S19 Fig). The 72,298 particles belonging to the first class were used for all the high-resolution 3D refinements. The full needle complex was reconstructed without imposing symmetry with local refinement and angular sampling 0.5°. Post-refinement was performed with relion_postprocess and consisted of the following steps: soft-masking the reconstruction, correction with high-noise substitution [64], correction for the modulation transfer function of the detector, FSC-based weighting and B-factor sharpening [20]. The final C1 reconstruction of the whole needle complex reaches 5.1 Å resolution as determined by the gold-standard procedure for FSC value 0.143 between two independent half data sets, and has been sharpened with B factor -162.

Focused refinement of the IM ring was performed imposing C24 symmetry with a solvent mask obtained from the C1 reconstruction. Angular sampling started from 1.8° and decreased stepwise to 0.2° during the process. The resulting map had resolution 3.9 Å and post-processing yielded a map with resolution 3.6 Å sharpened with B-factor -128. A second focused map was obtained including in the mask the IM ring and the connector regions and imposing C8 symmetry, which is common between the C24 fold symmetry of the IM ring and the C16 of the secretin, with angular sampling 0.9° and 0.5°. The map at 4.5 Å resolution was post-refined yielding 3.9 Å and sharpened with B-factor -120.

The high-resolution maps of the periplasmic IM ring and connector obtained applying C24 and C8 symmetry, respectively, allowed us to choose the correct handedness by modeling the structures of MxiG, MxiJ and the N-terminal domains of MxiD into them. The correct handedness of the other maps was established by comparing them with the former two maps.

### Model building and refinement

The map for periplasmic IM ring shows backbone and side chain features which allowed modeling of the atomic structure for MxiJ and the periplasmic domains of MxiG. We used Phyre2 [65] to generate homology models for them. The homology models were selected based on the X-ray crystal structure of the *Salmonella* homologues PrgH (PDB entry 3GR0) [26] and an EM Structure of PrgK (PDB entry 2Y9J) [21] due to their high sequence identity. The initial MxiJ model MxiJ_22-189_ was split into two domains, MxiJ_22-80_ and MxiJ_98-189_. MxiG_172-340,_ MxiJ_22-80_ and MxiJ_98-189_ were fitted as rigid body in the periplasmic ring of the focused C24 map low-pass filtered at 3.2 Å IM reconstruction with the software Chimera version 1.11.2 [66] and refined using Coot 0.8.8 [67] and phenix.real_space_refine release 2998 [68] applying NCS-like constraints between identical chains and secondary structure restraints. Manual building of the linker between the two MxiJ domains, the MxiJ N-and C-terminus and the MxiG N-terminus, followed by phenix refinement, resulted in MxiJ_21-197_ and MxiG_152-340_. Phenix CC_mask is 0.83, the EM ringer score [69] is 3.02.

The connector region is formed by the two N-terminal domains of MxiD and the C-terminal domain of MxiG. An initial model of the MxiD_37-171_ was built using the *Escherichia coli* EscC structure (PDB entry 3GR5) [26] as templates and fitted into the focused C8 map low-pass filtered at 3.5 Å using the fitmap tool of Chimera 1.13.1. The C-terminal domains of two out of three MxiG subunits were built *de novo* with Coot 0.8.9.1 using the bulky aromatic side chains as sequence markers. The entire connector model was refined with Coot and phenix.real_space_refine 3357 imposing secondary structure restraints and NCS-like constraints corresponding to C8 symmetry. Phenix CC_mask is 0.85 and EMringer score is 3.50 [69].

To build a model of the export apparatus core, the cryo-EM map of the flagellar export apparatus from *Salmonella* (EMDB entry 4173) [31] was initially fitted into the C1 map of the needle complex with the Chimera fitmap global search tool using the metric ‘correlation about the mean’ as readout. 100,000 searches were clustered into 84,840 unique fits. The best three fits have correlation values 0.3063, 0.2751 and 0.2646; p value calculated and corrected as described [70] are 1.88e-11, 5.74e-9 and 6.55e-8. All of them are therefore significant owing to the helical symmetry of the complex, and we chose the best fit to build the atomic model. Homology models for the export apparatus proteins SpaP, SpaQ and SpaR of *Shigella* were generated with SWISS-MODEL [71] using the subunits of the *Salmonella* flagellar export apparatus as templates (PDB entry 6F2D) [31]. We fitted them as rigid body in the map, using the fitted flagellar map as guide, and added an additional SpaQ subunit because corresponding helical features are visible in our map. Chimera fitmap global search was used also for initial location of the inner rod subunits. We used the structure of the *Shigella* needle filament (PDB entry 2MME) [37] as search model after transforming MxiH into polyalanine peptide and removing the N-terminal 10 non-helical residues. After visual inspection of the top fits, we chose the one filling all the unoccupied helical densities close to the export apparatus and removed the helical segments not supported by density, or nearby density that could be ascribed to the helices after flexible fitting. We kept 22 subunits, corresponding to four helical turns. Flexible fitting was performed on the whole system (export apparatus core and inner rod) in the map low-pass filtered at 6 Å with iMODFIT 1.44 [72] including all the atoms and using 15% of the normal modes. Afterwards, we removed residues 20–62, 28–45 and 36–46 from the three lowest SpaQ subunits, since no density supports them, and we introduced the MxiH side chain into the two upper turns of the inner rod. Finally, we minimized the system with one macrocycle of phenix.real_space_refine 3409, imposing secondary structure restraints, after low-pass filtering the map at 6 Å. Phenix CC_mask value is 0.62.

The atomic models of the IM ring, connector, export apparatus core and inner rod were validated as described [73] by comparison of the FSC curves between masked map generated from the atomic model and the cryo-EM half maps. Their resolution was estimated at FSC = 0.5 between masked map of the model and the full cryo-EM map. FSC curves were calculated with phenix.mtriage [74].

The OM ring map does not show distinct secondary structure features, therefore we did not attempt flexible fitting. We built a homology models for the N3 and secretin domains of MxiD using SWISS-MODEL and the recently published structure of the *Salmonella* open secretin pore [33]. We used the Chimera fitmap global search to place the two domains independently in the C1 map.

For the secretin domain homology model, 500,000 global searches with Chimera fitmap yielded 270,138 unique fits. The top 15 fits have correlation in the range 0.4453–0.3849 (the 16^th^ 0.3261) and adjusted p value 3.5e-12 [70]. As expected, they are located along the density that forms the double walled β-barrel in *Salmonella*. Each secretin domain includes residues 301–521.

For the N3 domain, 500,000 searches were clustered in 59,357 fits. Among the top 30 fits we found 9 poses in the expected position and conformation with respect to the map and the secretin domains (correlation 0.3732–0.3176). The small dimension of this domain and low quality of the C1 map on the tilted side of this region prevents the global search from finding the correct poses in the top hits. Thus, we modelled the remaining N3 domains by superposing one of the secretin-N3 subunits found by Chimera to the lonely secretin domains. Each N3 domain includes residues 179–230 and 259–300. The C-terminal S domain has not been modelled as explained in the main text.

### Structure analysis and representation

For the multiple sequence alignment, a structural alignment between MxiG/PrgH and MxiJ/PrgK was generated with PDBeFold [75] and orthologue sequences were added based on the Pfam alignment families PF09480 and PF01514. Clustal Omega [76] was used for aligning the last 12 amino acids. Multiple Sequence alignment figures were generated with ESPript [77].

Local resolution maps were calculated with Relion 2.1. Electrostatic analysis was carried out with APBS [78] using two-step focusing to solve the linearized Poisson-Boltzmann equation with multiple Debye-Hückel spheres as boundary conditions, 150 mM ionic strength and 37°C temperature. Interface analysis was done in Coot and Chimera and with the EBI PISA server [79]. Transmembrane predictions are based on results of the TMpred [80] and TMHHM pred server [81], Palymitoylation site predictions were carried out using PROSITE [82]; secondary structure predictions for MxiI are based on the PSIPRED server results [83].

Maps were segmented and edited with Segger inside Chimera [84] and masks were created using Relion.

The Channel analysis was performed using CHEXVIS [85] with a probe radius of 1.4 Å. The electrostatic potential at the central line is based on the APBS results for the IM ring.

Calculation of helical parameters of the export apparatus core is based on the centre of mass of SpaP subunits and has been performed with HELFIT [86]. SpaP has been superposed to SpaR and used to calculate the centre of mass of the sixth helical subunit.

Hydrophobicity of the protein surfaces was depicted following the Eisenberg scale [87].

RMSD of structural alignments has been calculated with the protein structure comparison service PDBeFold version 2.59 at the European Bioinformatics Institute (http://www.ebi.ac.uk/msd-srv/ssm) [75] with default parameters.

All molecular structure figures were generated with PyMol [88] or UCSF Chimera [66], developed by the Resource for Biocomputing, Visualization, and Informatics at the University of California, San Francisco, with support from NIH P41-GM103311.

### Site directed mutagenesis

To generate the MxiG mutants, the following primers and their reverse complements were used: 5´CTGTCAGAGAAGAACTGACAAAGGAAAAGCTTGAGCTC3´ for D311K, 5’GGTGTCAAACAAAGAAATAAATGAGATTCAACAATATATCAATC3’ for R208E, 5’GGTGTCAAACAAAGAAATAAATGCAATTCAACAATATATCAATC3’ for R208A, 5’ CTGGTGTCAAACAAAAGAATAAATAGAATTCAACAATATATC3’ for E205R, 5’ CTGGTGTCAAACAAAGCAATAAATAGAATTCAACAATATATCAATC3’ for E205A, 5’ GTTGAATTTCCGTATTTCAAAAATATTAAA3’ for Y263F. 5’ GATGATGATTTTTAAGGTAAATCATATC3’ for 347-Stop. The polymerase chain reaction was performed on MxiG encoded on the pASK-IBA5plus vector with a Phusion High-Fidelity DNA Polymerase. The PCR product was Dpn1 digested, and transformed in *E*. *coli* DH5α for selection and amplification. Sanger sequencing was used to verify mutagenesis and plasmids harboring the mutation were transformed in *Shigella* M90T ΔMxiG.

### Secretion assay and expression test

The secretion assay was performed as published [89] inducing the expression of MxiG and MxiG mutants in *Shigella* M90T ΔMxiG strains, encoded on a pASK-IBA5plus plasmid. For the secretion assay and the expression test, the sample cell density was normalized, lysed and analyzed by a SDS-PAGE gel or Western Blot. The Western Blot was performed using a polyvinylidene fluorid membrane with anti-IpaB, anti-IpaC and anti-DnaK (Enzo) antibodies. For the expression test, a western blot with a polyvinylidene fluorid membrane was performed using anti-DnaK (StressGene Biotechnologies Corp.) and anti–MxiG antibodies.

### Immunoflourescence imaging

To analyze the co-localization of the T3SS needle with the bacterial membrane, *Shigella* M90T ΔMxiG strains were co-transformed with MxiG mutants encoded on a pASK-IBA5plus plasmid and Strep-MxiH on a pASK-IBA3C plasmid. For co-localization studies of the MxiG mutants in the bacterial membrane, *Shigella* M90T ΔMxiG was transformed with MxiG mutants encoded on a pASK-IBA5plus plasmid only and treated with 0.1% Triton X-100 in Dubelcco´s—Phosphate buffered Saline (DPBS) and 10% w/v Lysozyme, 5 mM EDTA in DPBS at room temperature to permeabilize the membrane. Bacterial cells were fixed for 15’ at RT with 4% paraformaldehyde, followed by membrane permeabilization, if required. Staining was performed with a monoclonal primary mouse anti-Strep-tag II antibody (StrepMAB-Classic, IBA) in combination with an Alexa Flour 647 secondary antibody (Invitrogen). The bacterial membrane was stained with a DiO lipophilic tracer (Invitrogen). Bacteria were mounted in Mowiol 4–88 medium on 33 mm μ-dishes (ibidi) and visualized on a Leica SP8 confocal microscope equipped with a 63x, N.A. 1.40 oil immersion objective using excitation at 471 nm and 653 nm and operated in HyVolution mode. Deconvolution was performed with Huygens Essential (Huygens Compute Engine 18.04.0p7 64b) software using express deconvolution and standard profile. Experiments were performed multiple times on biological duplicates.

## Supporting information

S1 Fig Micrographs and 2D class averages of the needle complex. Representative micrograph of purified *Shigella* needle complexes (left) and class averages obtained in the final round of 2D classification (right).
(TIF)

S2 Fig Gold-standard FSC curves of the needle complex reconstructions. FSC curves from gold-standard refinement and post-refinement. The vertical line indicates the resolution of the maps at 0.143 FSC. (**A**) Full reconstruction without symmetry, resolution 5.1 Å. (**B**) Focused reconstruction of the IM ring and connector with C8 symmetry, resolution 3.9 Å. (**C**) Focused reconstruction of the IM ring with C24 symmetry, resolution 3.6 Å.
(TIF)

S3 Fig Local resolution maps of the 3D reconstructions. Cross-sections of the maps obtained in this study, locally low-pass-filtered and colored according to the local resolution (Å) as shown in the color scales. (**A**) Full reconstruction of the needle complex without symmetry. (**B**) Focused reconstruction of the IM ring and connector with C8 symmetry. (**C**) Focused reconstruction of the IM ring with C24 symmetry.
(TIF)

S4 Fig Density map and model-map FSC plot of the IM ring. (**A** and **B**) Detail of the C24 density map of the IM ring with the atomic model represented as sticks. Selected residues close the channel exit or involved in MxiG-MxiJ interaction and subjected to mutational analysis are labeled (Fig 4D and S7 Fig). (**C**) Model-map FSC curves of the IM ring. Model versus the full map used for building and refinement (black line), model refined against the first of the two independent half maps versus the same map (green, FSC_work_) and versus the second half map (blue, FSC_test_). The line at FSC 0.5 marks the approximate resolution of ~3.5 Å of the model.
(TIF)

S5 Fig Details of the polar MxiG-MxiJ and MxiG-MxiG interactions. Residues involved in polar interactions are depicted as sticks and labeled; dotted lines represent hydrogen bonds and salt bridges. (**A** and **B**) Interface between two adjacent MxiJ (shades of orange) and one MxiG subunit (blue). Interface MxiJ D2 –MxiG D4 (A). Interface MxiJ–MxiG D2 (B). **(C)** Interface between two adjacent MxiG subunits, involving the D3 and D4 domains.
(TIF)

S6 Fig Multiple sequence alignment of T3SS IM ring proteins from different species. MxiG and MxiJ protein sequences were aligned with orthologues from other Gram negative bacteria expressing T3SS. The highlighted secondary structure elements correspond to the *Shigella* proteins. Blue boxes indicate conserved residues with regards to their physicochemical properties. Fully conserved residues are depicted in white letters on red background. Residues involved in intermolecular interactions are marked with +, x or O. The green boxes indicate the MxiJ loops pointing in the direction of the export apparatus. MxiJ orthologues of the following species were aligned (UniProt code in brackets): *Shigella flexneri* (Q06081), *Salmonella typhimurium* (P41786), *Candidatus regiella insecticola* (E0WTJ1), *Sodalis glossinidius* (Q2NVJ4), *Burkholderia pseudomallei* (Q3JL03), *Chromobacterium violaceum* (Q7NUV9), *Yersinia enterocolitica* (Q01251) and *Pseudomonas aeruginosa* (Q9I314). All proteins mentioned belong to the SPI-1 family, except for Pseudomonas PscJ, which is part of the Ysc family. MxiG orthologues of the following species are aligned: *Shigella flexneri* (P0A221), *Salmonella typhimurium* (P41783), *Candidatus regiella insecticola* (G2H2F2), *Sodalis glossinidius* (Q2NR71), *Burkholderia pseudomallei* (Q63K19), Chromobacterium violaceum (Q7NVC0).
(TIF)

S7 Fig Secretion assay, expression test and localization of MxiG mutants. (**A**) The MxiG D311K mutant does not secrete effector proteins. Secreted proteins of wild type M90T strain (WT) and mutant strain were precipitated and visualized by coomassie stained SDS-PAGE. Protein secretion was induced adding Congo red (CR). SepA serves as a T3S-independent loading control. IpaA, IpaB, IpaH and IpaC are T3SS effectors. (**B**) Western blot of *Shigella* total cell lysates stained with antibodies against MxiG and DnaK for wild type M90T and MxiG mutants. (**C**) Secretion assay of the MxiG Y263F, Y263F_E205R and 347-Stop mutants. Secreted proteins and total cell lysates of wild type M90T strain (WT) and mutant strains were separated via SDS-PAGE and effector proteins (IpaB and IpaC) visualized by Western Blot; DnaK served as loading control. The effector proteins IpaB and IpaC are produced but not secreted in the MxiG mutant strains 347-Stop, Y263F and Y263F_E205R. (**D**) Representative immunofluorescence images of *Shigella* M90T ΔMxiG strains complemented with the Strep-MxiG mutants E205R and Y263F_E205R. The bacterial membrane was permeabilized prior to antibody staining. Strep-MxiG localizes at the bacterial membrane in both mutants. Red: anti-Strep antibody; green: lipophilic membrane dye. The scale bar corresponds to 2 μm.
(TIF)

S8 Fig Details of the MxiJ-MxiJ interaction. **(A** and **B)** Interface between adjacent MxiJ subunits in cartoon representation; residues involved in polar interactions are depicted as sticks and are labeled; dotted lines represent hydrogen bonds and salt bridges. (**A**) Polar interactions involving residues of the D1 domains. **(B)** Polar contacts at the MxiJ–MxiJ interface in the D2 domain. Only conserved or conservatively substituted residues involved in hydrogen bonds or salt bridges are depicted. **(C)** MxiJ surface involved in homo-interaction colored according to the Eisenberg hydrophobicity scale (white residues being most hydrophobic). Hydrophobic residues at the D2-D2 domain interface are labeled.
(TIF)

S9 Fig Electrostatic potential of the export apparatus core and MxiJ loops. **(A)** Side view of the outer surface of our model of the export apparatus core colored according to the electrostatic potential (scale +/- 5 kT/e^-^). Two MxiJ and two MxiG subunits (orange and blue, respectively) located on opposite sides of the IM ring are shown as cartoon. Two MxiJ loops are in close proximity to the export apparatus. The tip residues of the lower loop (residues 91–100, black box with continuous line) are conserved and interact mostly with the hydrophobic surface of SpaQ, while the residues of the upper loop (residues 133–144, box with dashed line) interact mostly with the more hydrophilic surface of SpaP. **(B)** Vertical cutaway of the export apparatus core showing the inner surface colored according to the electrostatic potential (scale +/- 5 kT/e^-^). The inner chamber is mostly positively charged. The bottom opening is closed, preventing substrate access, while the upper opening provides connection to the channel of the inner rod.
(TIF)

S10 Fig The IM ring channels of the *Shigella* and *Salmonella* T3SS. **(A)** Top view of a quarter of the IM ring with MxiG and MxiJ models in sky blue and orange cartoon representation, respectively. The IM channel surface is depicted in yellow. The radial channels connecting the periplasm with the export cage are interconnected by a circular channel. (**B**) Radial channel in the IM ring of *Salmonella* (PDB ID 5TCP) with PrgH and PrgK subunits in purple and beige cartoon representation, respectively. The channel is depicted in yellow, the exits A and B are labeled. (**C**) Radius and electrostatic potential along the central line of the *Salmonella* IM ring radial channel.
(TIF)

S11 Fig Validation of the connector model and its interface with the IM ring. **(A)** Model-map FSC curves of the connector (MxiD34-171 and MxiG338-367). Model versus the full map used for building and refinement (black line), model refined against the first of the two independent half maps versus the same map (green, FSC_work_) and versus the second half map (blue, FSC_test_). The line at FSC 0.5 marks the approximate model resolution of ~ 3.7 Å. **(B)** Detail of the β-sheet augmentation of MxiD_69-74_ with MxiG_362-371_ (left) and of the density map for MxiG_363-371_ (right). MxiD and MxiG carbon atoms are green and blue, respectively. Hydrogen bonds are depicted as dotted lines, the donor-acceptor distance indicated in Angstrom. **(C)** Slice through the focused C8 map and the cartoon models of IM ring and connector (right) and detail of the region between them (left). MxiG is depicted in blue, MxiJ in orange, MxiD in green. The MxiG C-terminal stretch connects IM ring and connector; the unassigned density between them could be occupied by the C-terminus of an adjacent MxiG subunit, which does not participate in the β-sheet augmentation.
(TIF)

S12 Fig Multiple sequence alignment of T3SS secretins from different species. Multiple sequence alignment of MxiD with orthologous from different T3SS expressing Gram-negative bacteria. Numbering and secondary structure elements of MxiD are indicated. Blue boxes highlight conserved residues with regards to their physicochemical properties. Identical residues are depicted in white letters on red background. MxiD orthologues of (UniProt code in brackets) *Shigella flexneri* (Q04641), *Salmonella typhimurium* (P35672), *E*.*coli EPEC* (B7UMB3), *Yersinia enterocolitica* (Q7BRZ9), and *Pseudomonas aeruginosa* (P95431) were aligned.
(TIF)

S13 Fig Polar and hydrophobic intramolecular interactions of the MxiD N0 and N1 domains. **(A**) N0 and N1 domains of MxiD colored from N- to C-terminus from blue to yellow (left) and close-up of the polar interaction (right). Residues forming hydrogen bonds are depicted as sticks and labeled. The side chains of Y96, Y105 and D136 bind backbone atoms of L134, A157 and K97, respectively **(B)** Tilted top view of the N0 and N1 domains of MxiD colored according to Eisenberg hydrophobicity scale (white residues being most hydrophobic). Residues involved in hydrophobic N0-N1 interactions are labeled and depicted as sticks (I94, W95, Y96, Y103, Y105 in N0 and L134, Y139, P155, P156, A157, L158 in N1).
(TIF)

S14 Fig Intermolecular interactions involving the MxiD and InvG N1 domain. **(A)** Two neighboring MxiD connector subunits (left). N1 residues involved in polar interaction are represented as sticks and labeled in the close-up (right). Black dotted lines represent N1-N1 interface hydrogen bonds (N144 –D131, T149 –L168, T149 –Y128, D147 –Y124, D147 –K171), yellow dotted lines hydrogen bonds between the N0 and N1 domains (R138 –N57, R142 –D98, R142 –N100, Y151 –D98, S153 –N100) **(B)** Two neighboring MxiD N1 domains colored according to the Eisenberg hydrophobicity scale (white residues being more hydrophobic) (left). Residues at the hydrophobic interface are depicted as sticks and labeled in the close-up (right) (A132, L134, Y128, L161, L168, L169 on the α-helices side and V114, L116 and Y151 on the β-sheet side). **(C)** N0 and N1 domains of two neighboring InvG subunits of the *Salmonella* connector (PDB ID 6DV3) (left); residues involved in polar intermolecular interactions represented as sticks and labeled in the close-up (right).
(TIF)

S15 Fig Surface properties of the *Shigella* needle and the OM pore. **(A**) Side view of the *Shigella* T3SS needle surface, colored according to its electrostatic potential (scale +/- 5 kT/e^-^). **(B)** Vertical cutaway of the OM ring, the surface colored as in (A). **(C)** Tilted top view of the surface of the open OM pore, colored according to the Eisenberg hydrophobicity scale (white representing hydrophobic areas). A ring of hydrophobic residues is visible on the membrane-facing side of the MA subdomain.
(TIF)

S16 Fig Cross-section of export apparatus, inner rod and needle and model-map FSC plot. **(A)** Vertical cross section of the semitransparent C1 map of the export apparatus, inner rod and start of the needle, with the protein subunits represented as cartoons (same color code as in Fig 7). The helices fit into regions of tubular-shaped density. **(B)** Model-map FSC curves. Model versus the full map used for building and refinement (black line), model refined against the first of the two independent half maps versus the same map (green, FSC_work_) and versus the second half map (blue, FSC_test_). The line at FSC 0.5 estimates the model resolution ~8 Å.
(TIF)

S17 Fig Comparison of distal SpaP subunits and secondary structure prediction of MxiI. **(A)** Subunits of the first inner rod turn (dark magenta and purple) and distal SpaP (light blue) of the *Shigella* needle complex superposed with the distal SpaP subunit of the isolated *Salmonella* flagellar system (dark blue). The N-terminal helix of the SpaP subunits occludes the opening between the export apparatus and the inner rod in the isolated flagellar system, while it is kinked and leaves the passage open in the *Shigella* needle complex. **(B)** Secondary structure prediction of MxiI generated with the PSIPRED server (http://bioinf.cs.ucl.ac.uk/psipred). Blue bars represent the confidence value. Pink tubes represent helices. C = coil, H = helix.
(TIF)

S18 Fig Cα RMSDs calculated from superposed orthologous protein domains in *Shigella* and *Salmonella*. The structure of the *Salmonella* domains has been obtained from the models with PDB ID 5TCP (PrgH, PrgK) or 6DV3 (InvG). Fraction of matched residues are relative to the *Shigella* domains.
(PDF)

S19 Fig Reference map used for the initial 3D classification. Reconstruction obtained from a preliminary dataset composed of ~3000 particle images collected on a FEI Tecnai Spirit microscope at 120 kV. The map was low-pass filtered at 60 Å before using it as reference for initial classification of the high-resolution dataset collected at the FEI Titan Krios.
(TIF)
