# Supplementary figures and images for: Cryo-EM structure of the Shigella type III needle complex
Source: PLoS Pathog. 2020 Feb 24;16(2):e1008263. doi: 10.1371/journal.ppat.1008263 (PMC7058355; doi:10.1371/journal.ppat.1008263)

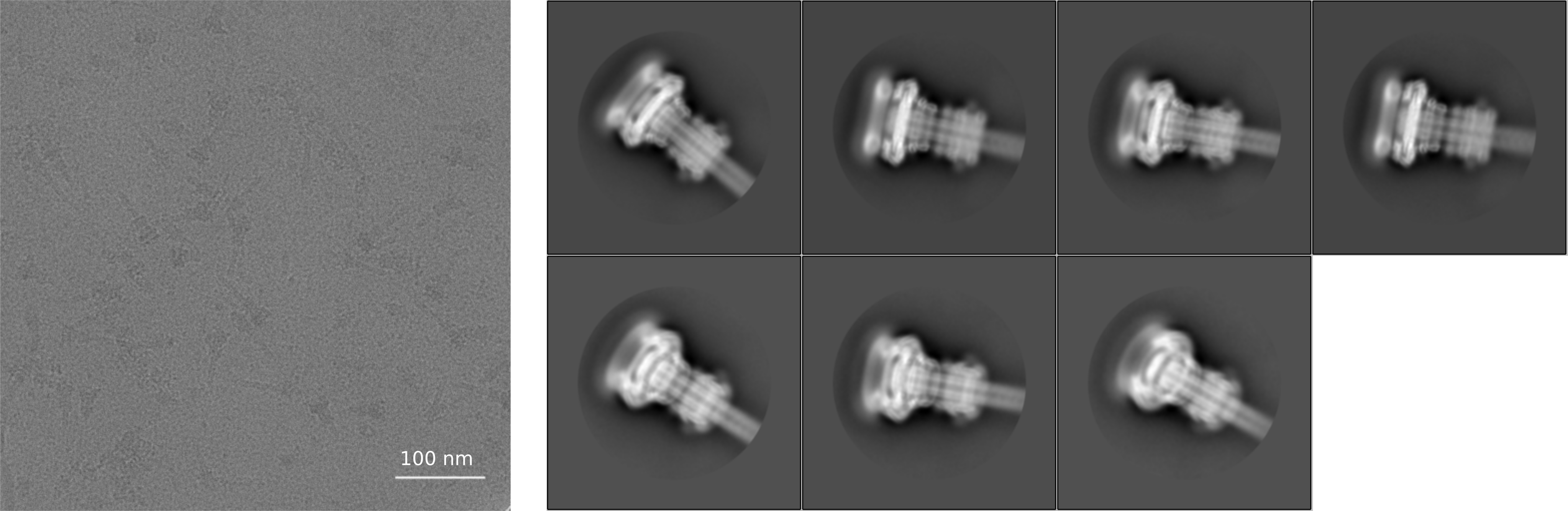

Supplement: S1 Fig — Representative micrograph of purified Shigella needle complexes (left) and class averages obtained in the final round of 2D classification (right). (TIF) [file ppat.1008263.s001.tif]

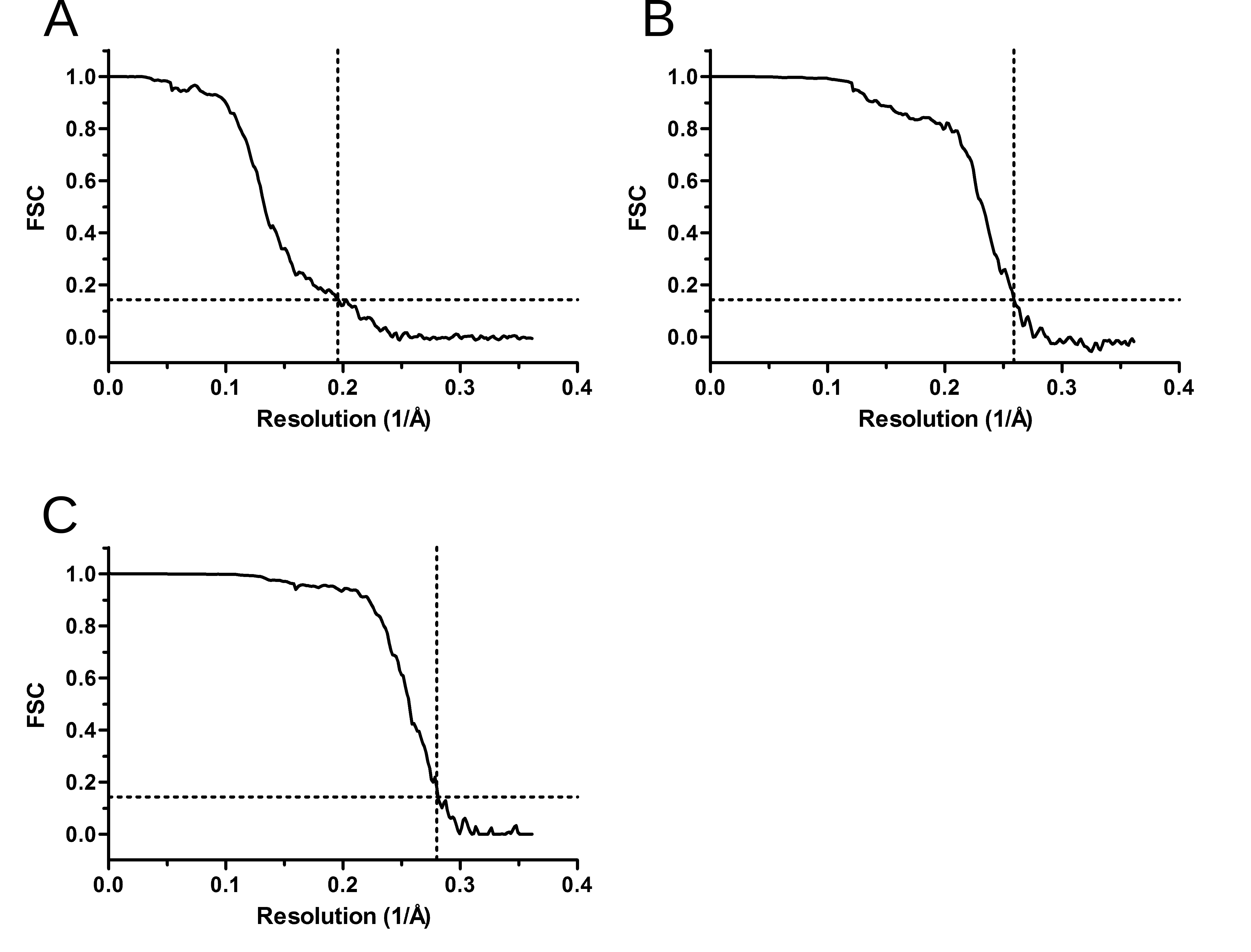

Supplement: S2 Fig — FSC curves from gold-standard refinement and post-refinement. The vertical line indicates the resolution of the maps at 0.143 FSC. (A) Full reconstruction without symmetry, resolution 5.1 Å. (B) Focused reconstruction of the IM ring and connector with C8 symmetry, resolution 3.9 Å. (C) Focused reconstruction of the IM ring with C24 symmetry, resolution 3.6 Å. (TIF) [file ppat.1008263.s002.tif]

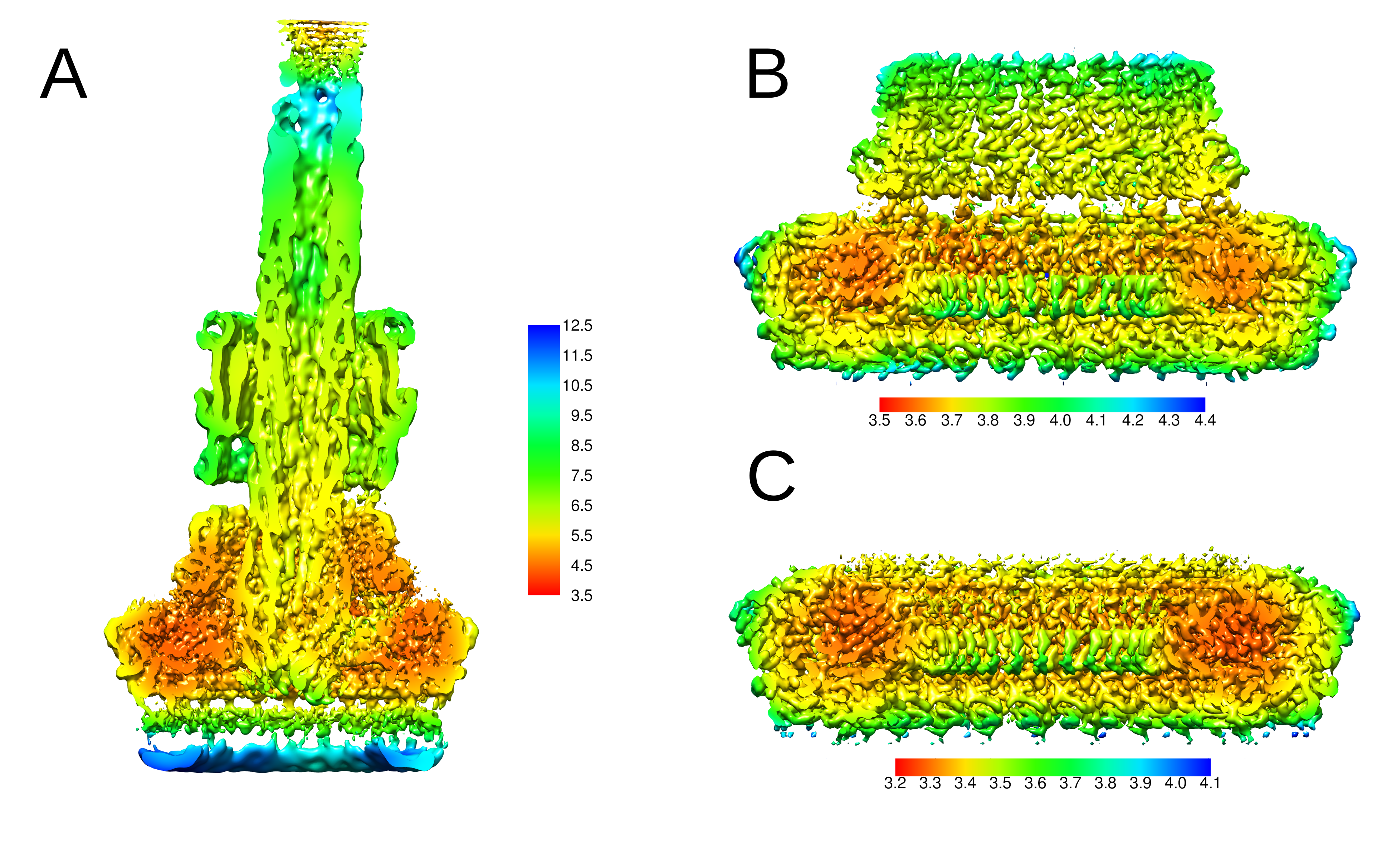

Supplement: S3 Fig — Cross-sections of the maps obtained in this study, locally low-pass-filtered and colored according to the local resolution (Å) as shown in the color scales. (A) Full reconstruction of the needle complex without symmetry. (B) Focused reconstruction of the IM ring and connector with C8 symmetry. (C) Focused reconstruction of the IM ring with C24 symmetry. (TIF) [file ppat.1008263.s003.tif]

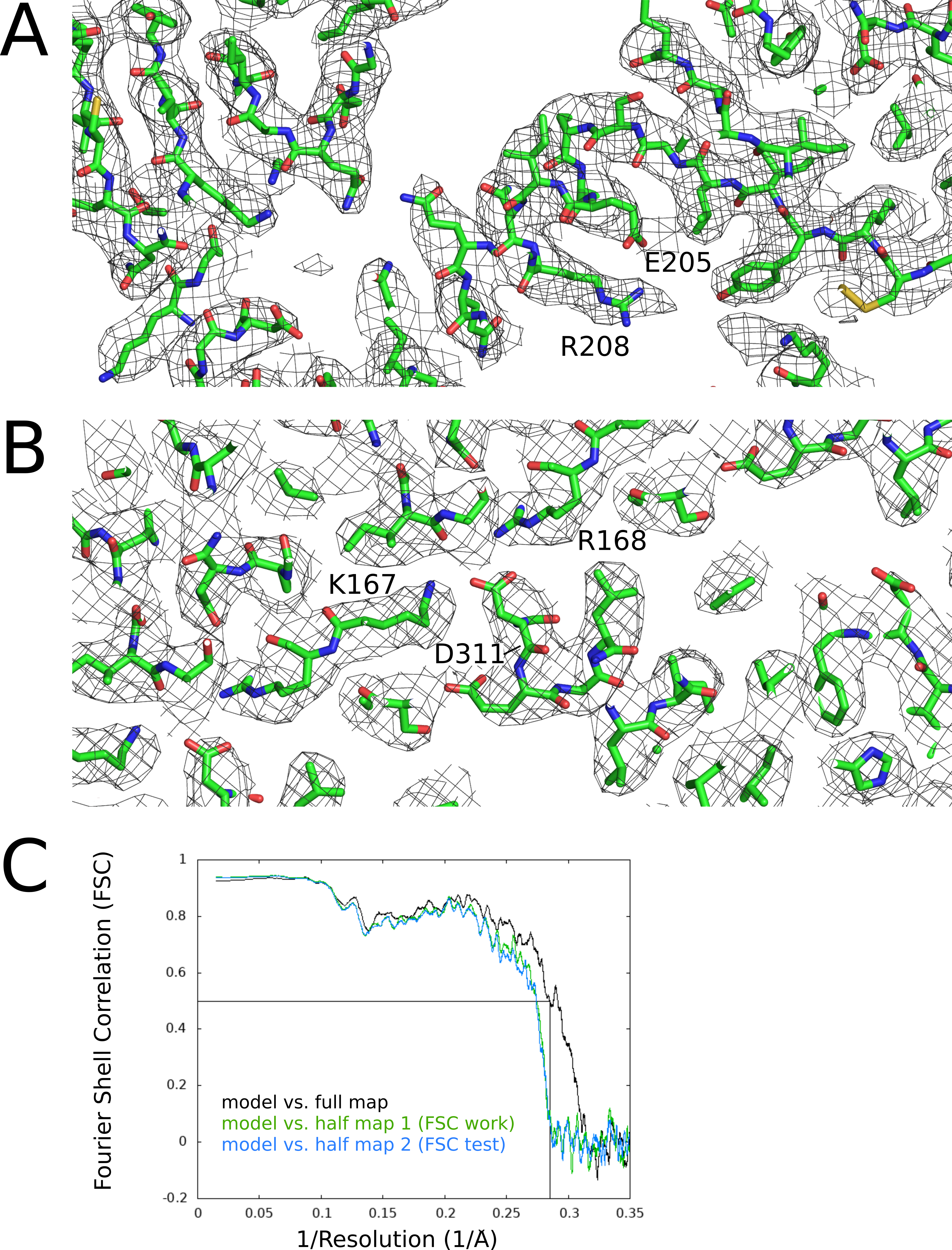

Supplement: S4 Fig — (A and B) Detail of the C24 density map of the IM ring with the atomic model represented as sticks. Selected residues close the channel exit or involved in MxiG-MxiJ interaction and subjected to mutational analysis are labeled (Fig 4D and S7 Fig). (C) Model-map FSC curves of the IM ring. Model versus the full map used for building and refinement (black line), model refined against the first of the two independent half maps versus the same map (green, FSCwork) and versus the second half map (blue, FSCtest). The line at FSC 0.5 marks the approximate resolution of ~3.5 Å of the model. (TIF) [file ppat.1008263.s004.tif]

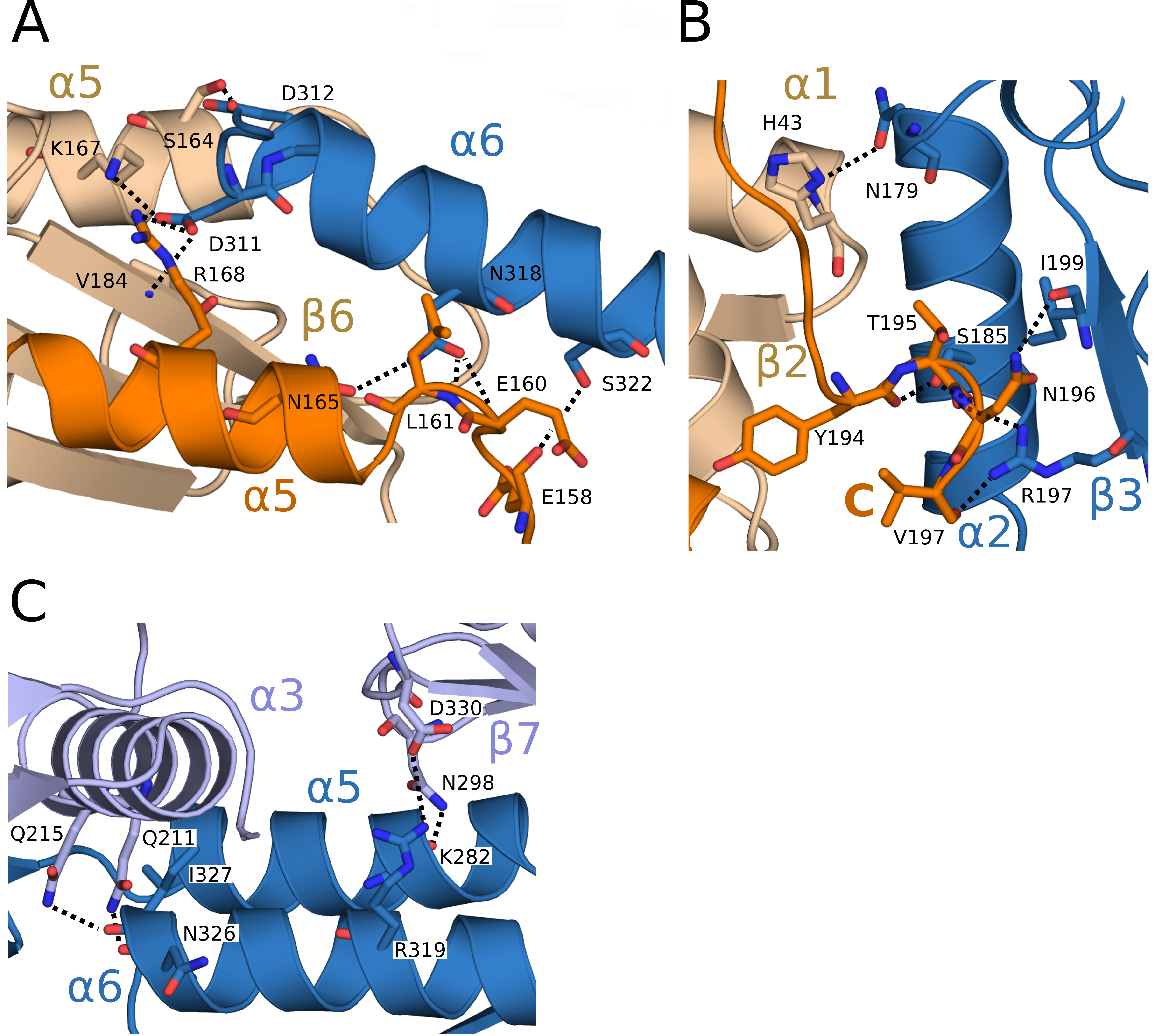

Supplement: S5 Fig — Residues involved in polar interactions are depicted as sticks and labeled; dotted lines represent hydrogen bonds and salt bridges. (A and B) Interface between two adjacent MxiJ (shades of orange) and one MxiG subunit (blue). Interface MxiJ D2 –MxiG D4 (A). Interface MxiJ–MxiG D2 (B). (C) Interface between two adjacent MxiG subunits, involving the D3 and D4 domains. (TIF) [file ppat.1008263.s005.tif]

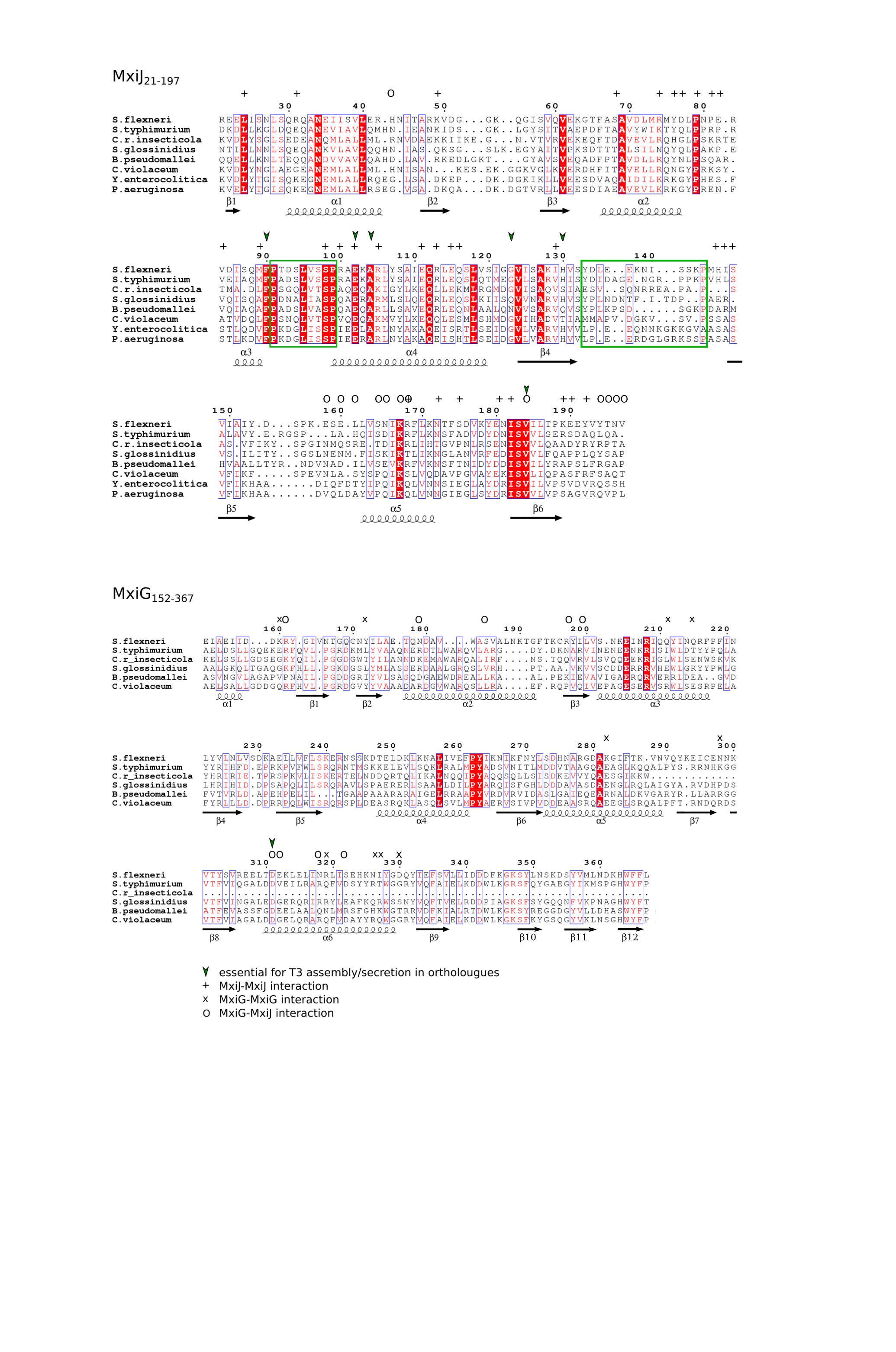

Supplement: S6 Fig — MxiG and MxiJ protein sequences were aligned with orthologues from other Gram negative bacteria expressing T3SS. The highlighted secondary structure elements correspond to the Shigella proteins. Blue boxes indicate conserved residues with regards to their physicochemical properties. Fully conserved residues are depicted in white letters on red background. Residues involved in intermolecular interactions are marked with +, x or O. The green boxes indicate the MxiJ loops pointing in the direction of the export apparatus. MxiJ orthologues of the following species were aligned (UniProt code in brackets): Shigella flexneri (Q06081), Salmonella typhimurium (P41786), Candidatus regiella insecticola (E0WTJ1), Sodalis glossinidius (Q2NVJ4), Burkholderia pseudomallei (Q3JL03), Chromobacterium violaceum (Q7NUV9), Yersinia enterocolitica (Q01251) and Pseudomonas aeruginosa (Q9I314). All proteins mentioned belong to the SPI-1 family, except for Pseudomonas PscJ, which is part of the Ysc family. MxiG orthologues of the following species are aligned: Shigella flexneri (P0A221), Salmonella typhimurium (P41783), Candidatus regiella insecticola (G2H2F2), Sodalis glossinidius (Q2NR71), Burkholderia pseudomallei (Q63K19), Chromobacterium violaceum (Q7NVC0). (TIF) [file ppat.1008263.s006.tif]

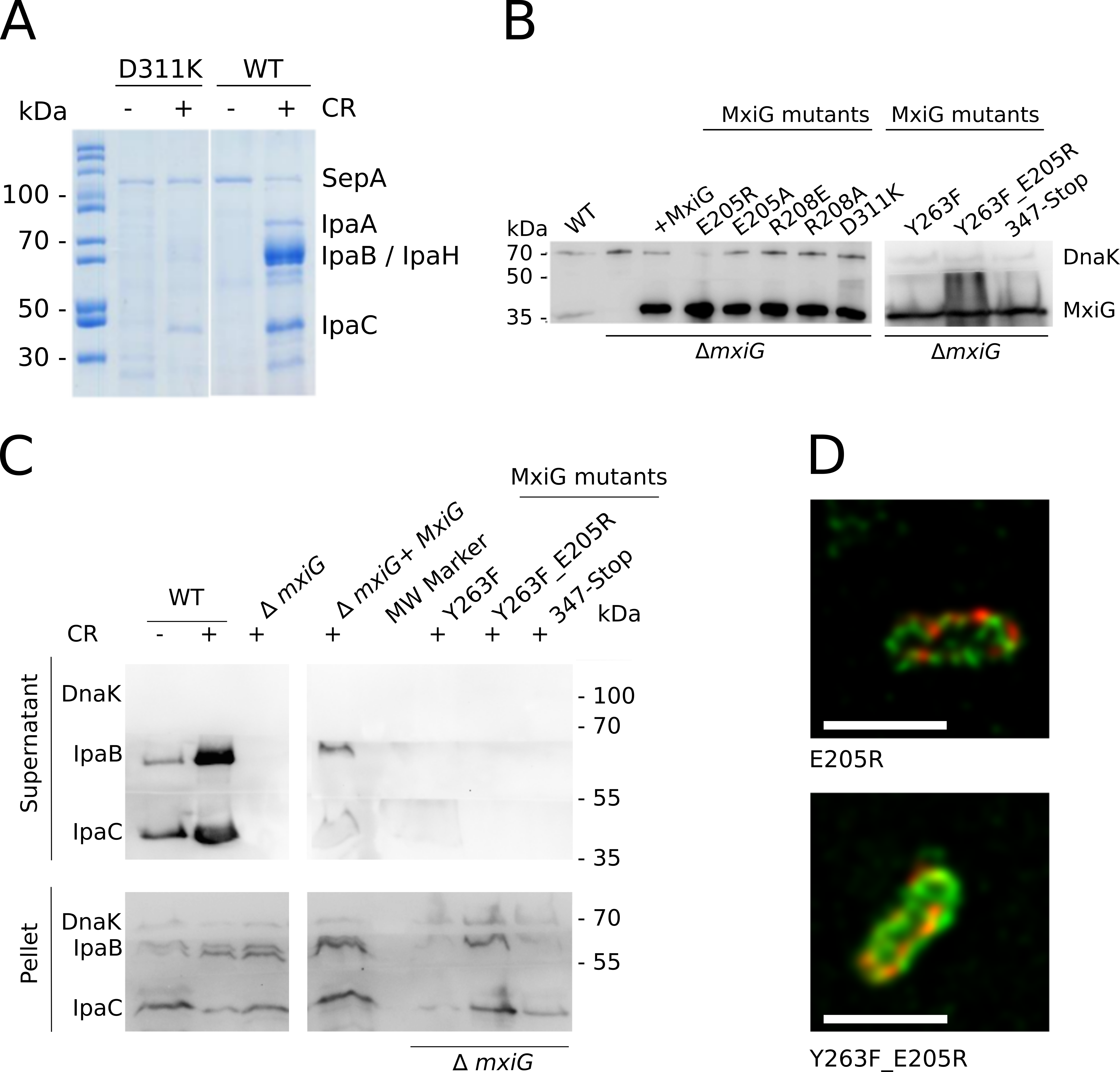

Supplement: S7 Fig — (A) The MxiG D311K mutant does not secrete effector proteins. Secreted proteins of wild type M90T strain (WT) and mutant strain were precipitated and visualized by coomassie stained SDS-PAGE. Protein secretion was induced adding Congo red (CR). SepA serves as a T3S-independent loading control. IpaA, IpaB, IpaH and IpaC are T3SS effectors. (B) Western blot of Shigella total cell lysates stained with antibodies against MxiG and DnaK for wild type M90T and MxiG mutants. (C) Secretion assay of the MxiG Y263F, Y263F_E205R and 347-Stop mutants. Secreted proteins and total cell lysates of wild type M90T strain (WT) and mutant strains were separated via SDS-PAGE and effector proteins (IpaB and IpaC) visualized by Western Blot; DnaK served as loading control. The effector proteins IpaB and IpaC are produced but not secreted in the MxiG mutant strains 347-Stop, Y263F and Y263F_E205R. (D) Representative immunofluorescence images of Shigella M90T ΔMxiG strains complemented with the Strep-MxiG mutants E205R and Y263F_E205R. The bacterial membrane was permeabilized prior to antibody staining. Strep-MxiG localizes at the bacterial membrane in both mutants. Red: anti-Strep antibody; green: lipophilic membrane dye. The scale bar corresponds to 2 μm. (TIF) [file ppat.1008263.s007.tif]

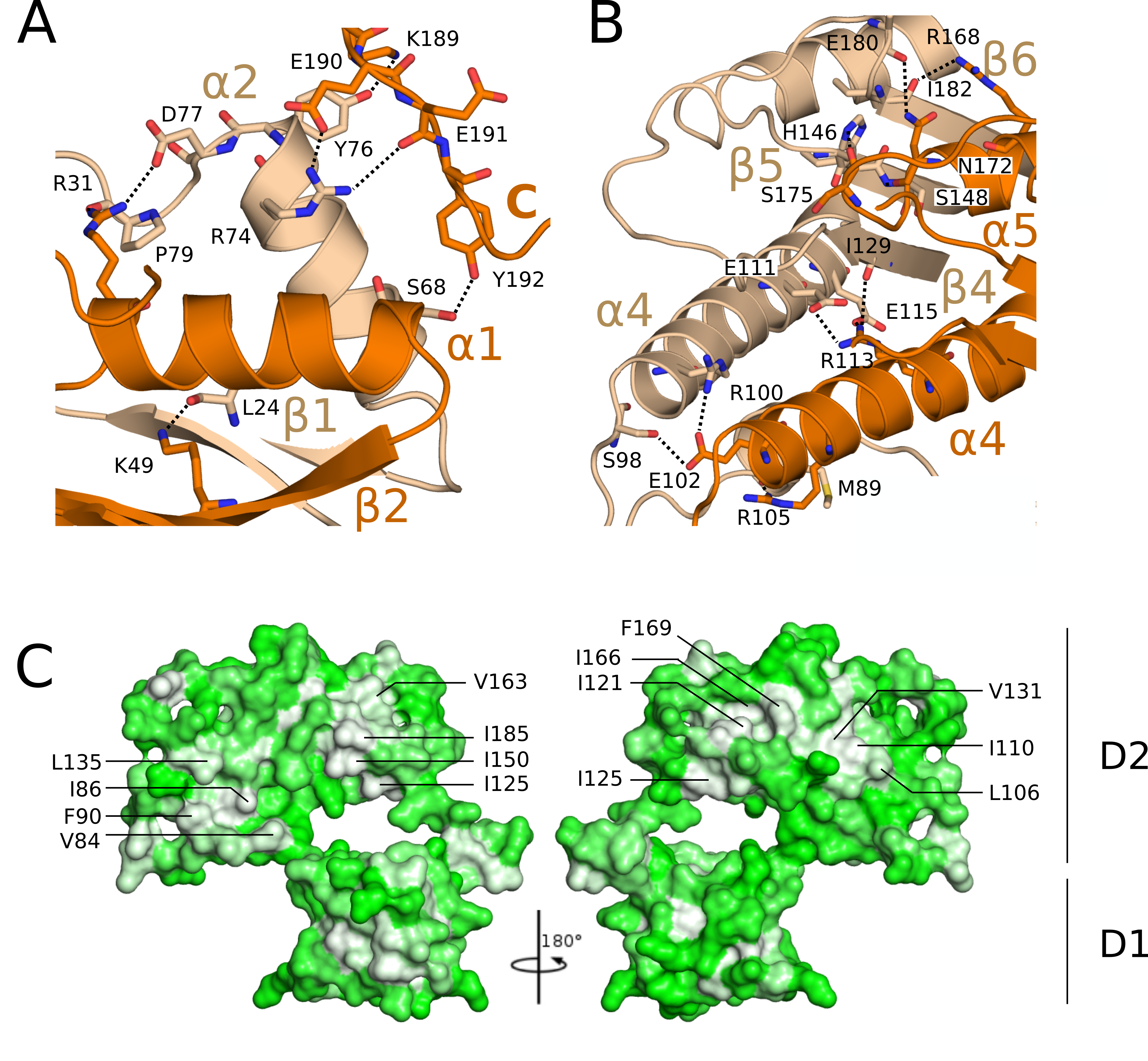

Supplement: S8 Fig — (A and B) Interface between adjacent MxiJ subunits in cartoon representation; residues involved in polar interactions are depicted as sticks and are labeled; dotted lines represent hydrogen bonds and salt bridges. (A) Polar interactions involving residues of the D1 domains. (B) Polar contacts at the MxiJ–MxiJ interface in the D2 domain. Only conserved or conservatively substituted residues involved in hydrogen bonds or salt bridges are depicted. (C) MxiJ surface involved in homo-interaction colored according to the Eisenberg hydrophobicity scale (white residues being most hydrophobic). Hydrophobic residues at the D2-D2 domain interface are labeled. (TIF) [file ppat.1008263.s008.tif]

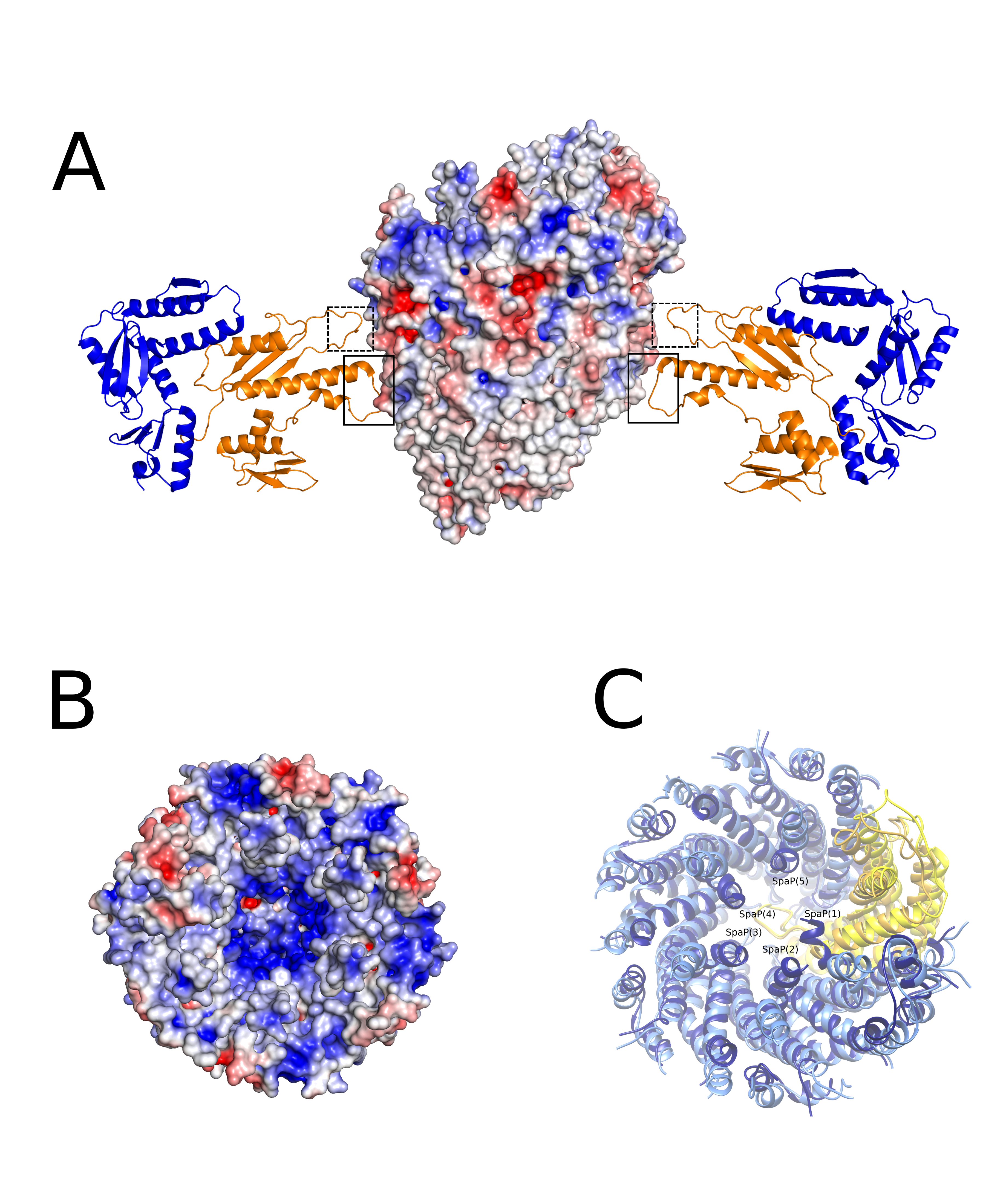

Supplement: S9 Fig — (A) Side view of the outer surface of our model of the export apparatus core colored according to the electrostatic potential (scale +/- 5 kT/e-). Two MxiJ and two MxiG subunits (orange and blue, respectively) located on opposite sides of the IM ring are shown as cartoon. Two MxiJ loops are in close proximity to the export apparatus. The tip residues of the lower loop (residues 91–100, black box with continuous line) are conserved and interact mostly with the hydrophobic surface of SpaQ, while the residues of the upper loop (residues 133–144, box with dashed line) interact mostly with the more hydrophilic surface of SpaP. (B) Vertical cutaway of the export apparatus core showing the inner surface colored according to the electrostatic potential (scale +/- 5 kT/e-). The inner chamber is mostly positively charged. The bottom opening is closed, preventing substrate access, while the upper opening provides connection to the channel of the inner rod. (TIF) [file ppat.1008263.s009.tif]

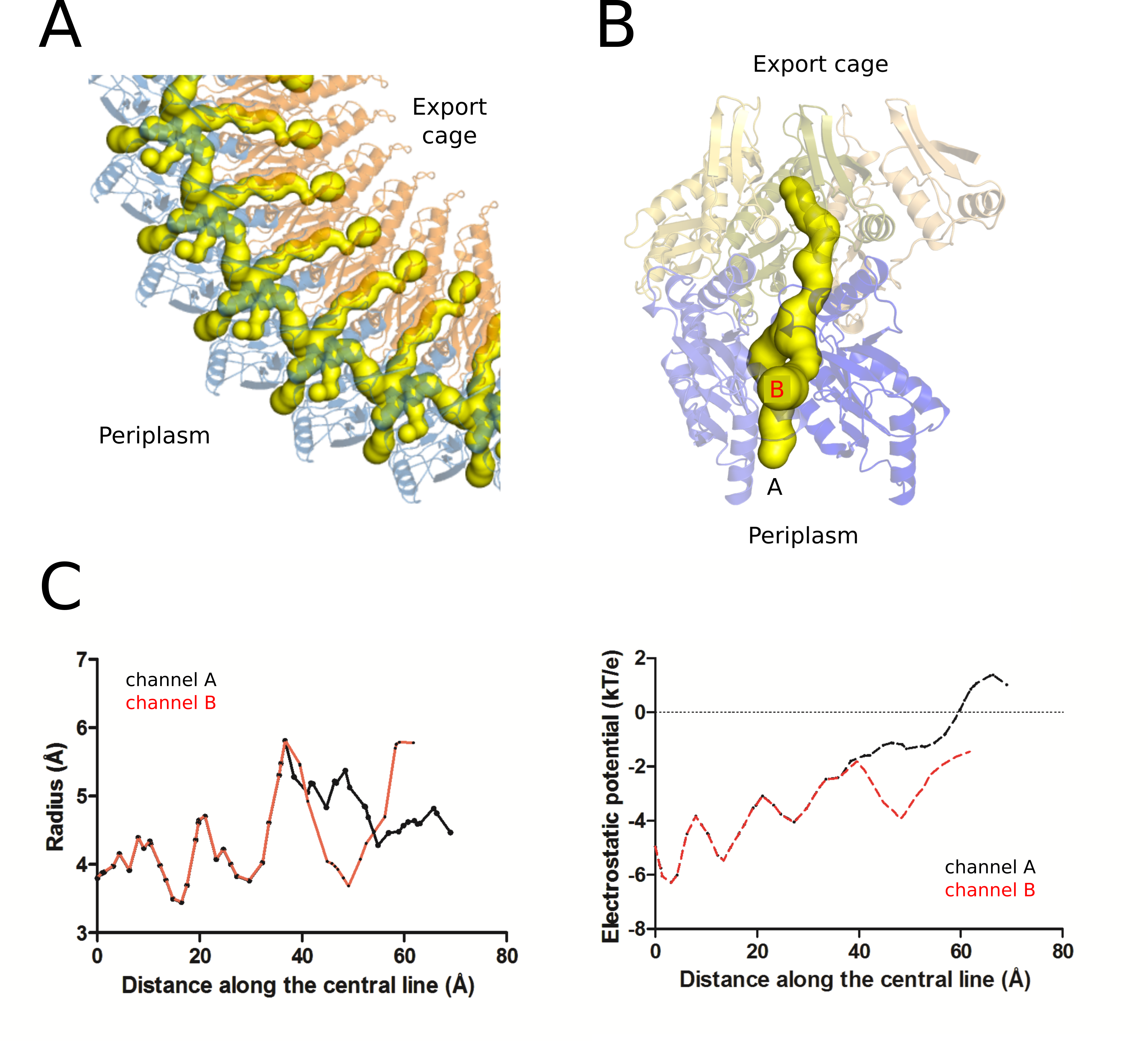

Supplement: S10 Fig — (A) Top view of a quarter of the IM ring with MxiG and MxiJ models in sky blue and orange cartoon representation, respectively. The IM channel surface is depicted in yellow. The radial channels connecting the periplasm with the export cage are interconnected by a circular channel. (B) Radial channel in the IM ring of Salmonella (PDB ID 5TCP) with PrgH and PrgK subunits in purple and beige cartoon representation, respectively. The channel is depicted in yellow, the exits A and B are labeled. (C) Radius and electrostatic potential along the central line of the Salmonella IM ring radial channel. (TIF) [file ppat.1008263.s010.tif]

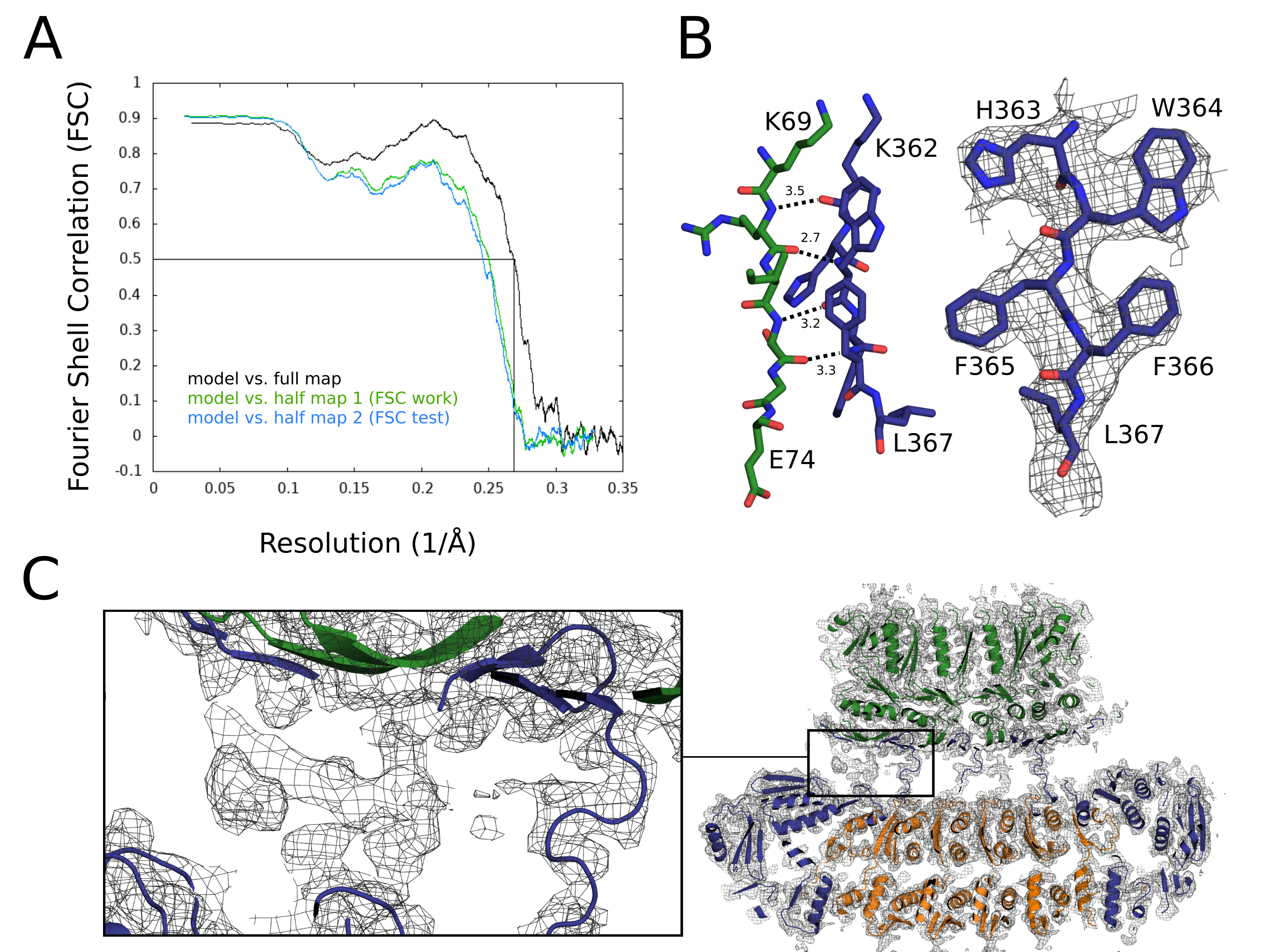

Supplement: S11 Fig — (A) Model-map FSC curves of the connector (MxiD34-171 and MxiG338-367). Model versus the full map used for building and refinement (black line), model refined against the first of the two independent half maps versus the same map (green, FSCwork) and versus the second half map (blue, FSCtest). The line at FSC 0.5 marks the approximate model resolution of ~ 3.7 Å. (B) Detail of the β-sheet augmentation of MxiD69-74 with MxiG362-371 (left) and of the density map for MxiG363-371 (right). MxiD and MxiG carbon atoms are green and blue, respectively. Hydrogen bonds are depicted as dotted lines, the donor-acceptor distance indicated in Angstrom. (C) Slice through the focused C8 map and the cartoon models of IM ring and connector (right) and detail of the region between them (left). MxiG is depicted in blue, MxiJ in orange, MxiD in green. The MxiG C-terminal stretch connects IM ring and connector; the unassigned density between them could be occupied by the C-terminus of an adjacent MxiG subunit, which does not participate in the β-sheet augmentation. (TIF) [file ppat.1008263.s011.tif]

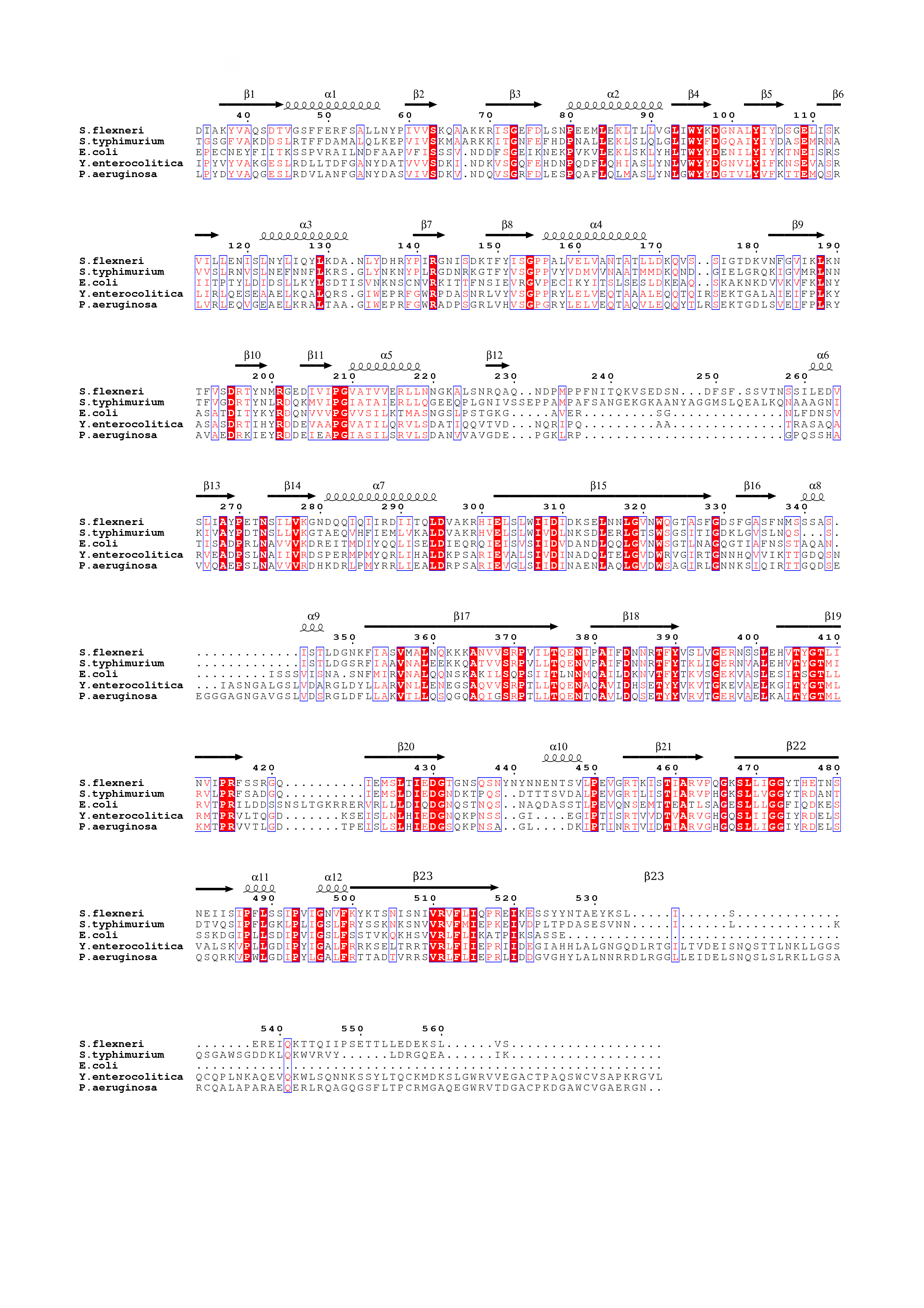

Supplement: S12 Fig — Multiple sequence alignment of MxiD with orthologous from different T3SS expressing Gram-negative bacteria. Numbering and secondary structure elements of MxiD are indicated. Blue boxes highlight conserved residues with regards to their physicochemical properties. Identical residues are depicted in white letters on red background. MxiD orthologues of (UniProt code in brackets) Shigella flexneri (Q04641), Salmonella typhimurium (P35672), E.coli EPEC (B7UMB3), Yersinia enterocolitica (Q7BRZ9), and Pseudomonas aeruginosa (P95431) were aligned. (TIF) [file ppat.1008263.s012.tif]

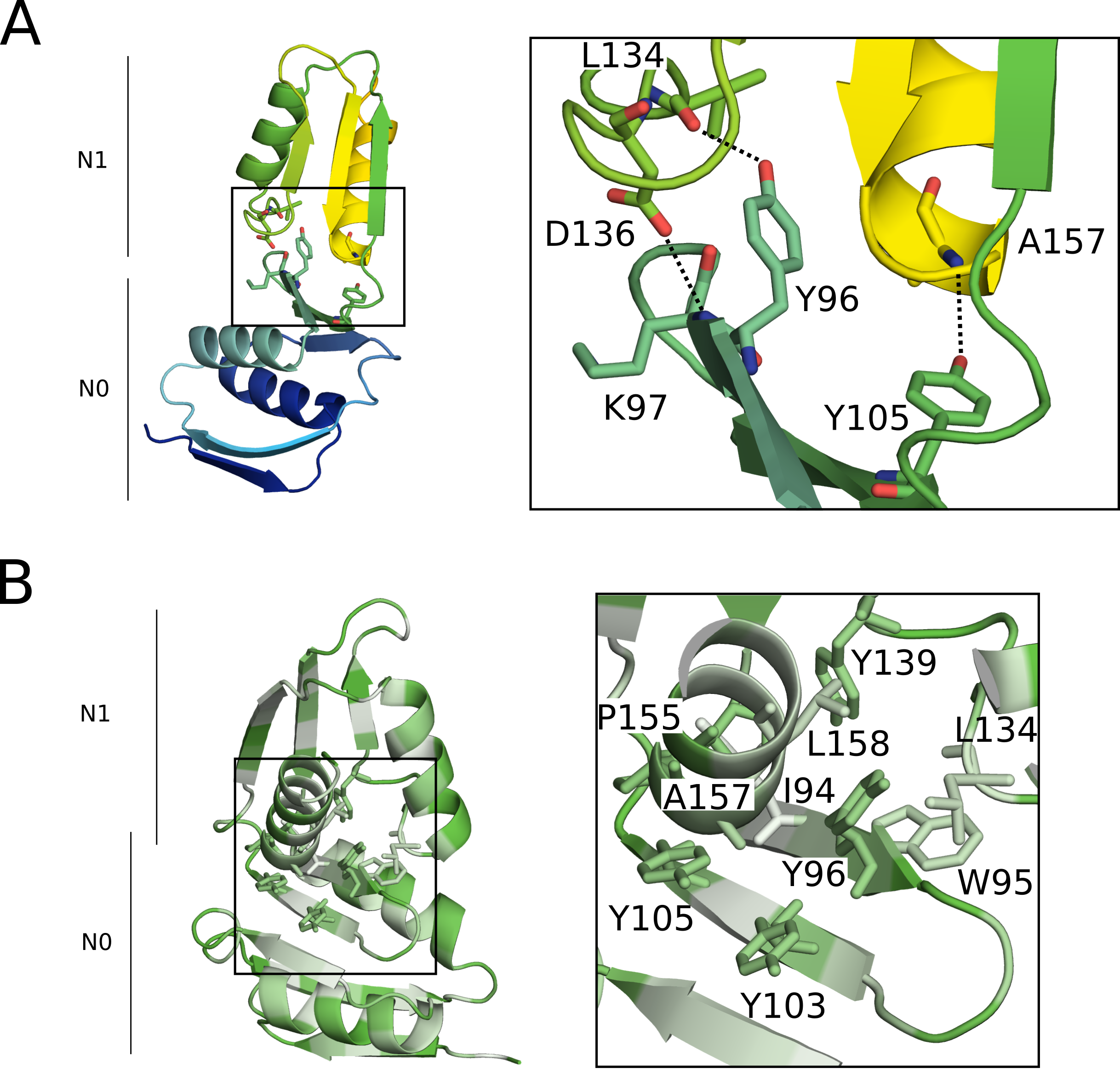

Supplement: S13 Fig — (A) N0 and N1 domains of MxiD colored from N- to C-terminus from blue to yellow (left) and close-up of the polar interaction (right). Residues forming hydrogen bonds are depicted as sticks and labeled. The side chains of Y96, Y105 and D136 bind backbone atoms of L134, A157 and K97, respectively (B) Tilted top view of the N0 and N1 domains of MxiD colored according to Eisenberg hydrophobicity scale (white residues being most hydrophobic). Residues involved in hydrophobic N0-N1 interactions are labeled and depicted as sticks (I94, W95, Y96, Y103, Y105 in N0 and L134, Y139, P155, P156, A157, L158 in N1). (TIF) [file ppat.1008263.s013.tif]

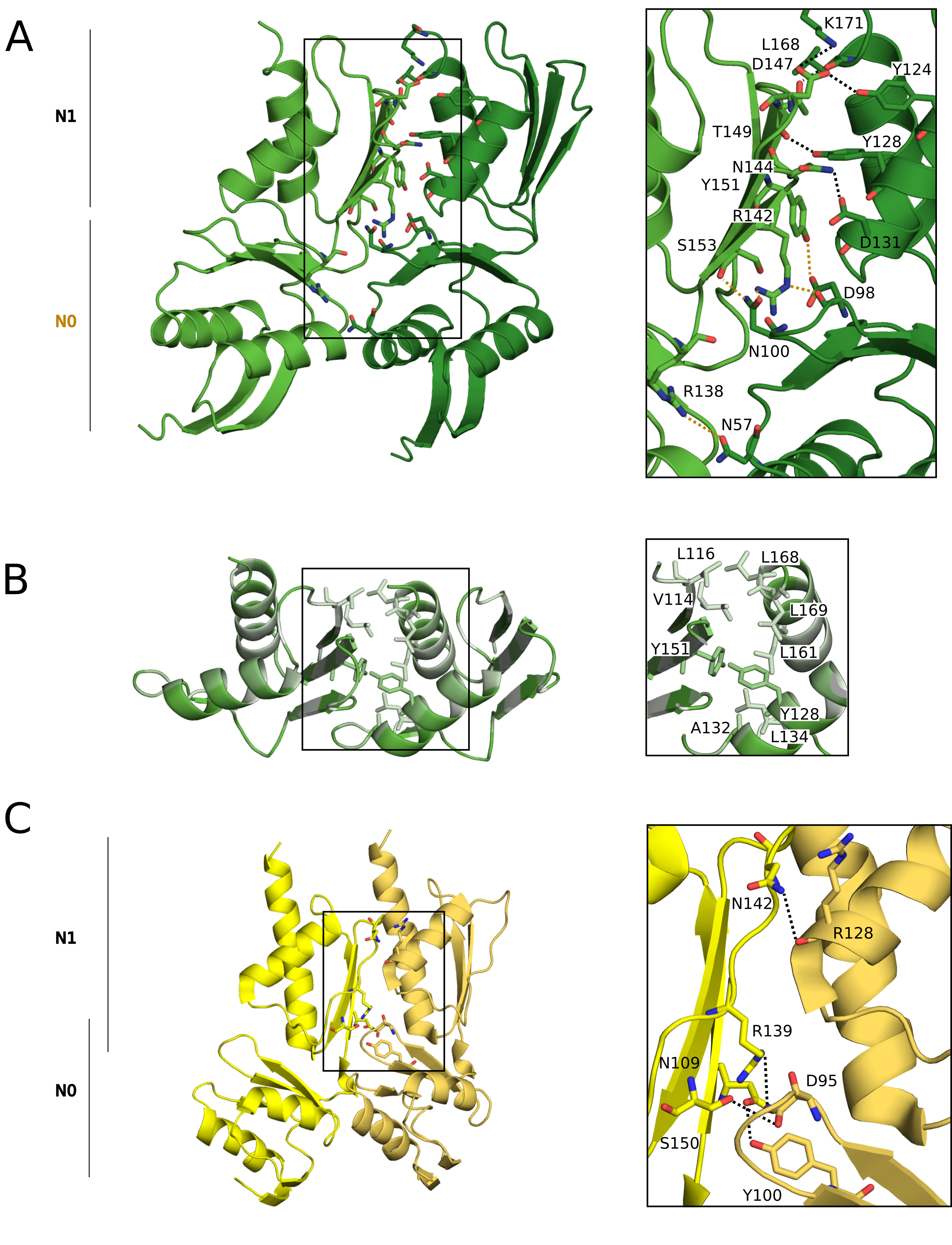

Supplement: S14 Fig — (A) Two neighboring MxiD connector subunits (left). N1 residues involved in polar interaction are represented as sticks and labeled in the close-up (right). Black dotted lines represent N1-N1 interface hydrogen bonds (N144 –D131, T149 –L168, T149 –Y128, D147 –Y124, D147 –K171), yellow dotted lines hydrogen bonds between the N0 and N1 domains (R138 –N57, R142 –D98, R142 –N100, Y151 –D98, S153 –N100) (B) Two neighboring MxiD N1 domains colored according to the Eisenberg hydrophobicity scale (white residues being more hydrophobic) (left). Residues at the hydrophobic interface are depicted as sticks and labeled in the close-up (right) (A132, L134, Y128, L161, L168, L169 on the α-helices side and V114, L116 and Y151 on the β-sheet side). (C) N0 and N1 domains of two neighboring InvG subunits of the Salmonella connector (PDB ID 6DV3) (left); residues involved in polar intermolecular interactions represented as sticks and labeled in the close-up (right). (TIF) [file ppat.1008263.s014.tif]

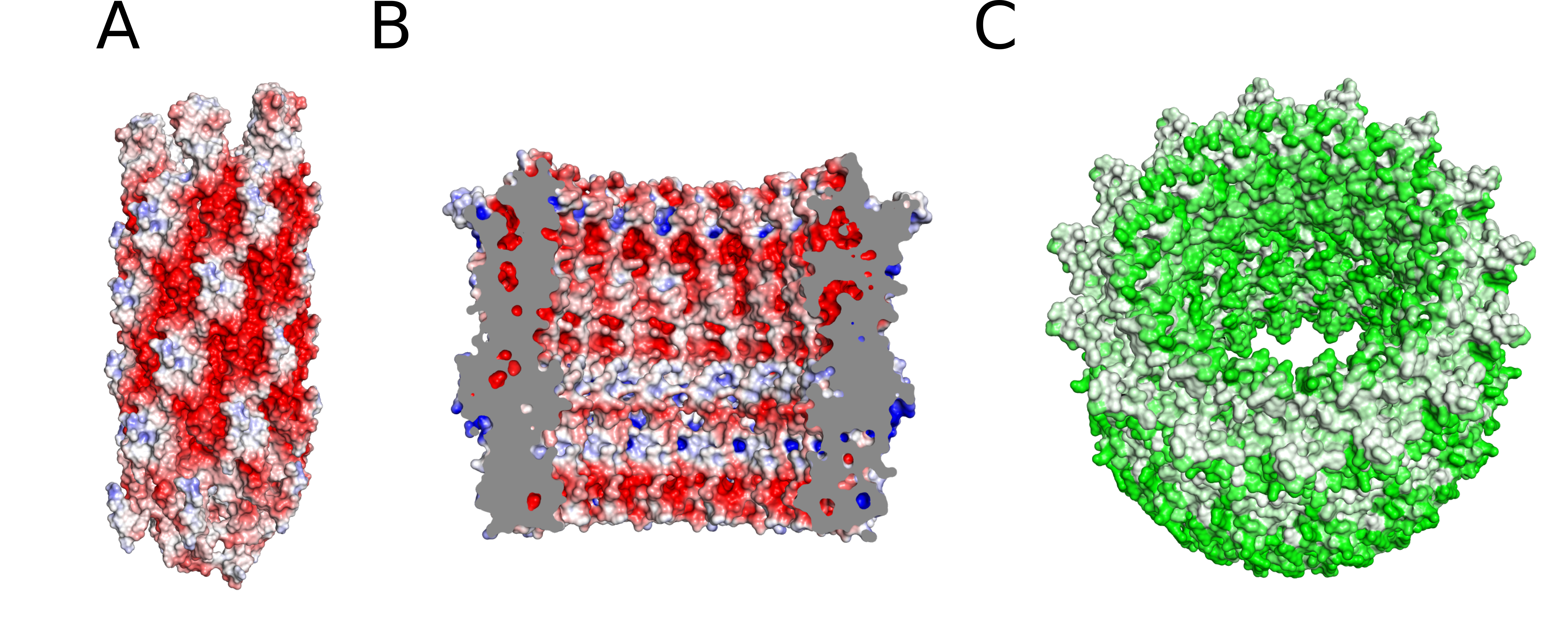

Supplement: S15 Fig — (A) Side view of the Shigella T3SS needle surface, colored according to its electrostatic potential (scale +/- 5 kT/e-). (B) Vertical cutaway of the OM ring, the surface colored as in (A). (C) Tilted top view of the surface of the open OM pore, colored according to the Eisenberg hydrophobicity scale (white representing hydrophobic areas). A ring of hydrophobic residues is visible on the membrane-facing side of the MA subdomain. (TIF) [file ppat.1008263.s015.tif]

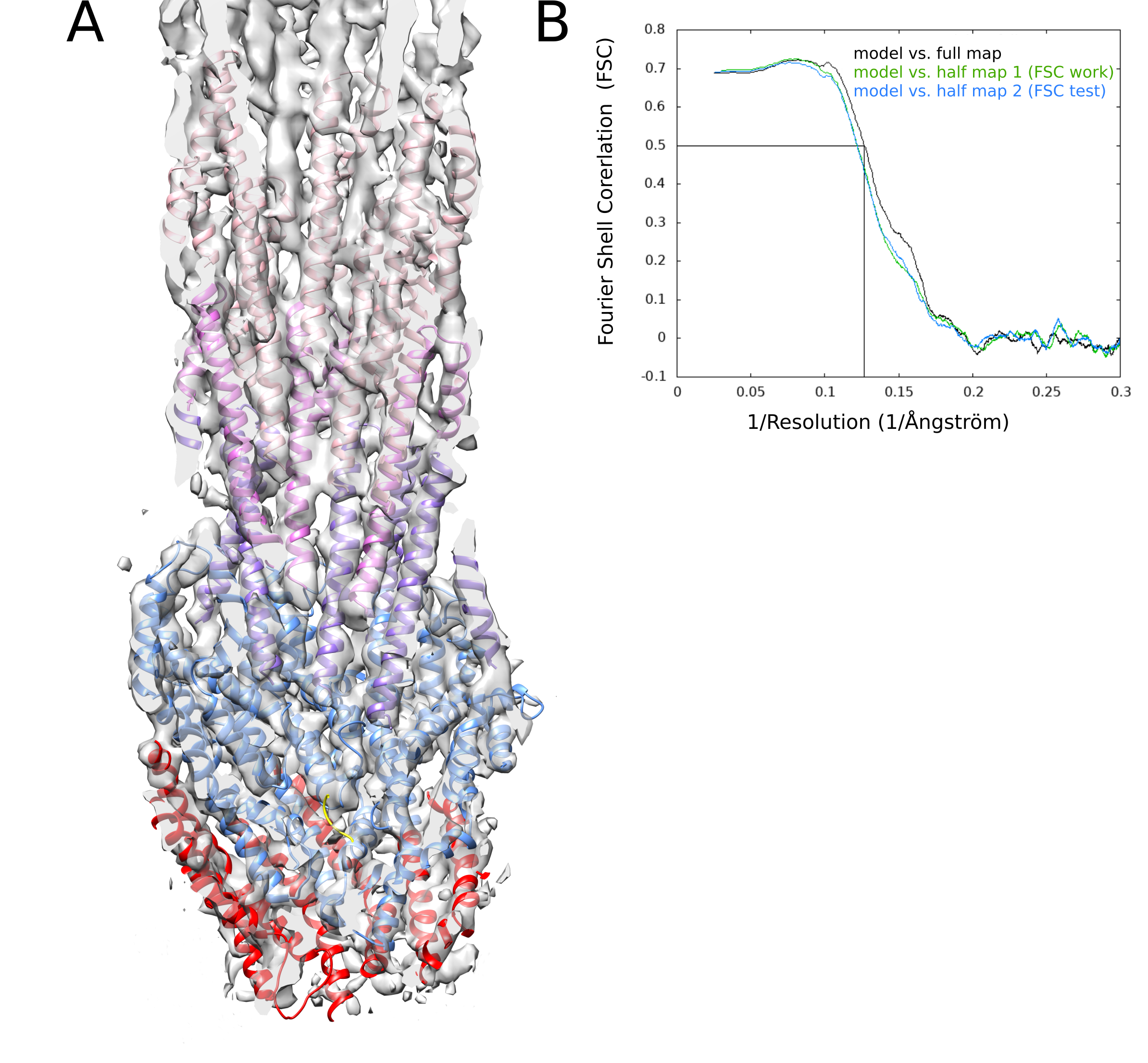

Supplement: S16 Fig — (A) Vertical cross section of the semitransparent C1 map of the export apparatus, inner rod and start of the needle, with the protein subunits represented as cartoons (same color code as in Fig 7). The helices fit into regions of tubular-shaped density. (B) Model-map FSC curves. Model versus the full map used for building and refinement (black line), model refined against the first of the two independent half maps versus the same map (green, FSCwork) and versus the second half map (blue, FSCtest). The line at FSC 0.5 estimates the model resolution ~8 Å. (TIF) [file ppat.1008263.s016.tif]

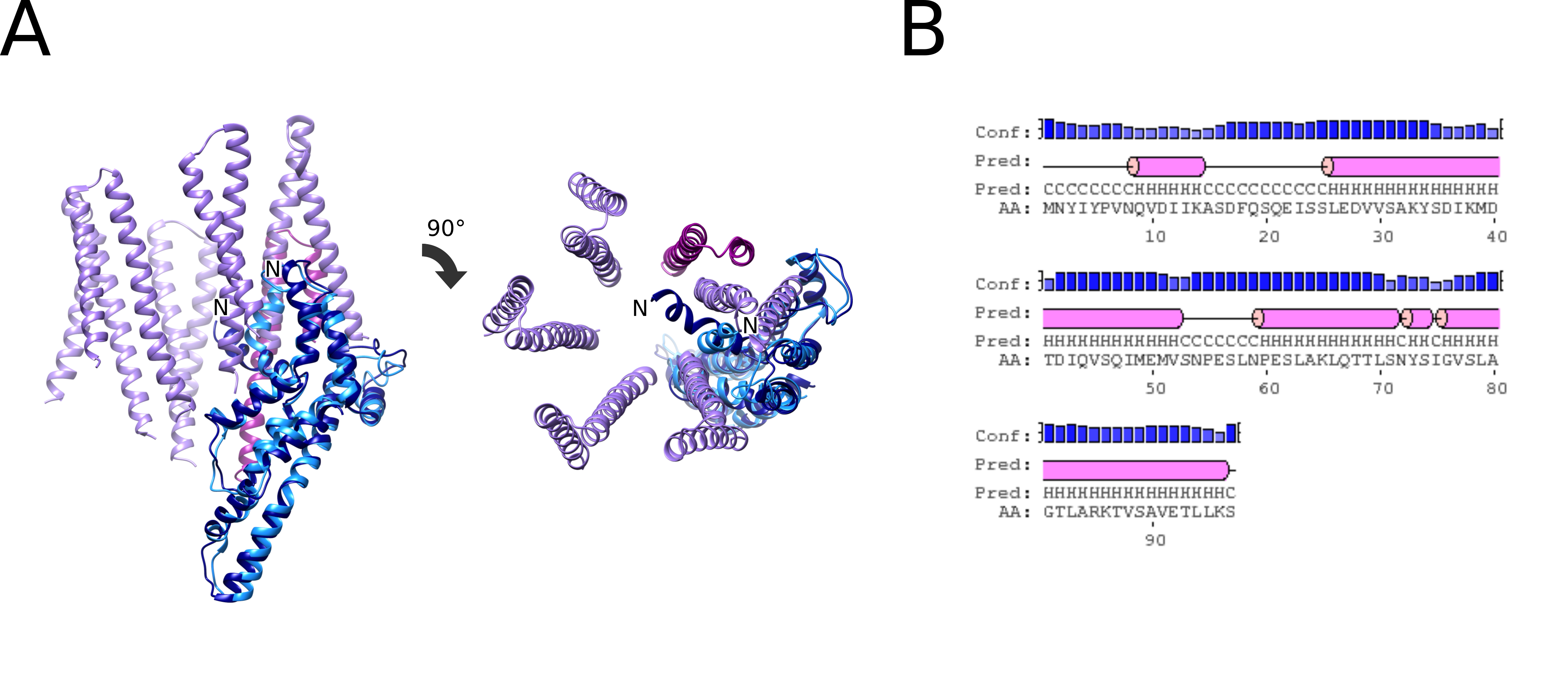

Supplement: S17 Fig — (A) Subunits of the first inner rod turn (dark magenta and purple) and distal SpaP (light blue) of the Shigella needle complex superposed with the distal SpaP subunit of the isolated Salmonella flagellar system (dark blue). The N-terminal helix of the SpaP subunits occludes the opening between the export apparatus and the inner rod in the isolated flagellar system, while it is kinked and leaves the passage open in the Shigella needle complex. (B) Secondary structure prediction of MxiI generated with the PSIPRED server (http://bioinf.cs.ucl.ac.uk/psipred). Blue bars represent the confidence value. Pink tubes represent helices. C = coil, H = helix. (TIF) [file ppat.1008263.s017.tif]

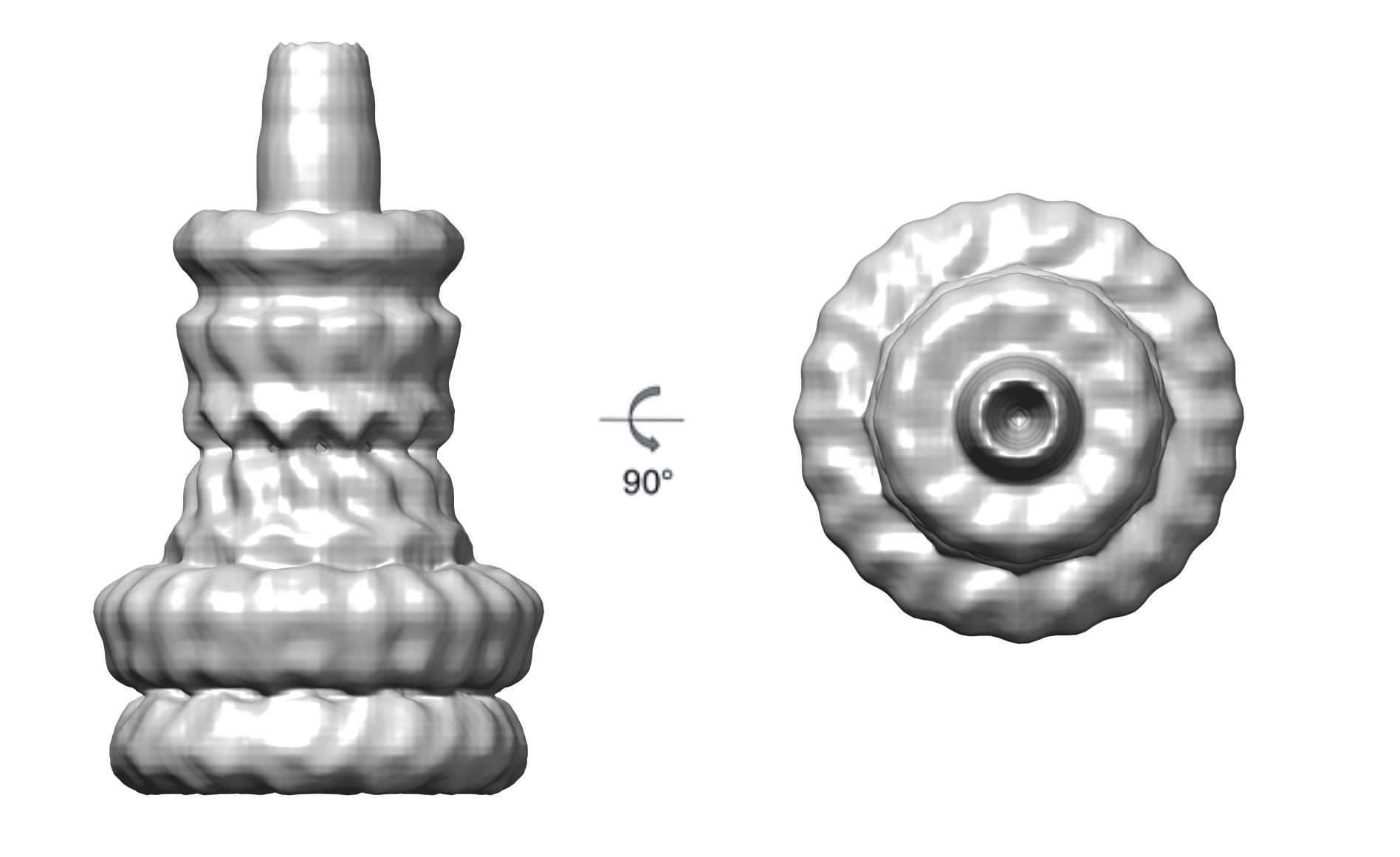

Supplement: S19 Fig — Reconstruction obtained from a preliminary dataset composed of ~3000 particle images collected on a FEI Tecnai Spirit microscope at 120 kV. The map was low-pass filtered at 60 Å before using it as reference for initial classification of the high-resolution dataset collected at the FEI Titan Krios. (TIF) [file ppat.1008263.s019.tif]
