# Supplementary material for: Cryo-EM structure of the Shigella type III needle complex
Source: PLoS Pathog. 2020 Feb 24;16(2):e1008263. doi: 10.1371/journal.ppat.1008263 (PMC7058355; doi:10.1371/journal.ppat.1008263)
Supplement: S18 Fig — The structure of the Salmonella domains has been obtained from the models with PDB ID 5TCP (PrgH, PrgK) or 6DV3 (InvG). Fraction of matched residues are relative to the Shigella domains. (PDF) [file ppat.1008263.s018.pdf]

Shigella	*Salmonella*	Domain	% Matched residues	RMSD (Å)
$MxiG_{152-200}$	$PrgH_{171-223}$	D2	98	1.26
$MxiG_{201-272}$	$PrgH_{224-294}$	D3	99	1.49
$MxiG_{273-340}$	$PrgH_{295-362}$	D4	100	1.33
$MxiJ_{21-77}$	$PrgK_{20-76}$	D1	100	0.82
$MxiJ_{97-191}$	$PrgK_{96-196}$	D2	95	1.25
$MxiD_{34-109}$	$InvG_{34-106}$	N0	93	1.62, 1.63*
$MxiD_{110-171}$	$InvG_{108-172}$	N1	94	2.45, 2.60*

S18 Fig. Cα RMSDs calculated from superposed orthologous protein domains in *Shigella* and *Salmonella*.

The structure of the *Salmonella* domains has been obtained from the models with PDB ID 5TCP (PrgH, PrgK) or 6DV3 (InvG). Fraction of matched residues are relative to the *Shigella* domains.

* Two RMSD values for the MxiD domains are reported because they have been refined with C8 symmetry.
